# Multi-Strategy Boosted Fick’s Law Algorithm for Engineering Optimization Problems and Parameter Estimation

**DOI:** 10.3390/biomimetics9040205

**Published:** 2024-03-28

**Authors:** Jialing Yan, Gang Hu, Jiulong Zhang

**Affiliations:** 1 Department of Applied Mathematics, Xi’an University of Technology, Xi’an 710054, China; yanjialing2019@sina.com; 2 Computer Network Information Center, Xi’an University of Technology, Xi’an 710048, China; zjl@xaut.edu.cn

**Keywords:** Fick’s law algorithm, differential variation, Gaussian local variation, comprehensive learning, seagull update strategy, engineering optimization

## Abstract

To address the shortcomings of the recently proposed Fick’s Law Algorithm, which is prone to local convergence and poor convergence efficiency, we propose a multi-strategy improved Fick’s Law Algorithm (FLAS). The method combines multiple effective strategies, including differential mutation strategy, Gaussian local mutation strategy, interweaving-based comprehensive learning strategy, and seagull update strategy. First, the differential variation strategy is added in the search phase to increase the randomness and expand the search degree of space. Second, by introducing the Gaussian local variation, the search diversity is increased, and the exploration capability and convergence efficiency are further improved. Further, a comprehensive learning strategy that simultaneously updates multiple individual parameters is introduced to improve search diversity and shorten the running time. Finally, the stability of the update is improved by adding a global search mechanism to balance the distribution of molecules on both sides during seagull updates. To test the competitiveness of the algorithms, the exploration and exploitation capability of the proposed FLAS is validated on 23 benchmark functions, and CEC2020 tests. FLAS is compared with other algorithms in seven engineering optimizations such as a reducer, three-bar truss, gear transmission system, piston rod optimization, gas transmission compressor, pressure vessel, and stepped cone pulley. The experimental results verify that FLAS can effectively optimize conventional engineering optimization problems. Finally, the engineering applicability of the FLAS algorithm is further highlighted by analyzing the results of parameter estimation for the solar PV model.

## 1. Introduction

Optimization algorithms are always asked to find optimal or near-optimal solutions [1]. Heuristic algorithms and meta-heuristic algorithms (Mas) are concrete implementations of optimization algorithms. Separately, heuristic algorithms optimize the performance of the algorithm by searching the solution space through heuristic rules and empirical guidance [2]. In addition, MAs ensure that the algorithm achieves optimal results by combining and adjusting different heuristic algorithms.

MAs are widely used and essential in science, technology, finance, and medicine. For example, in artificial intelligence (AI), MAs improve the model’s performance and accuracy [3]. It can also be used to optimize problems in AI applications such as intelligent recommendation systems, image processing, and natural language processing to provide better user experience and quality of service. In the financial field, MAs can be used to optimize investment portfolios and risk management to improve investment returns and reduce risks. In the medical field, the MAs can be used to optimize the allocation and scheduling of medical resources to optimize work efficiency in medical services. In transportation, MAs can be used to optimize traffic signal control and route planning to reduce traffic congestion and improve transportation efficiency [4] and so on. In addition, the application of MAs presents some opportunities for algorithmic improvement, multi-objective optimization, and integration with machine learning, but also some challenges, including high complexity, parameter selection, and local optimal solutions.

There are many different types of MAs. This paper mainly classifies them according to animal, plant, discipline, and other types. Animal-based optimization algorithms simulate the social behavior of animals in groups and collectives. For example, the best known is Particle Swarm Optimization (PSO), which simulates specific behaviors of birds during flight or foraging [5]. Krill Herd (KH) [6] simulates the grazing behavior of individual krill. Harris Hawk Optimization (HHO) [7] is mainly inspired by the cooperative behavior and chasing style of Harris hawks in nature. Abdel-Basset et al. proposed a Nutcracker Optimizer (NO) [8]. NO simulates two different behaviors exhibited by Nutcracker at different times. Whale Optimization Algorithm (WOA) [9] was developed, inspired by the feeding process of whales. The Genghis Khan Shark Optimizer (GKSO) simulates [10] predation and survival behaviors.

A plant-based optimization algorithm simulates the intelligent clustering behavior of plants. Dandelion Optimizer (DO) simulates the process of dandelion seeds flying over long distances relying on the wind [11]. Invasive Weed Optimization (IWO) simulates the basic processes of dispersal, growth, reproduction, and competitive extinction of weed seeds in nature [12]. Tree Growth Algorithm (TGA) [13] is an algorithm that simulates the competition among trees for access to light and food.

Discipline-based approaches include primarily chemical, mathematical, and physical approaches. They both accomplish optimization by simulating natural physical, fundamental laws, and chemical phenomena. Physics-based methods, such as those seen in Zhang et al., propose a Growth Optimizer (GO) subject to the learning and reflective mechanisms of individuals during social growth. It mathematically simulates growth behavior [14]. Abdel-Basset and El-Shahat proposed a Young’s Double-Slit Experiment (YDSE) optimizer. The YDSE optimizer was inspired by Young’s double-slit experiment [15]. Special Relativity Search (SRS) simulates the interaction of particles in a magnetic field [16]. Chemistry-based methods, such as Atomic Search Optimization (ASO), mathematically models and simulates the movement of atoms in nature [17]. Nature-inspired chemical reaction optimization algorithms mimic the principles of chemical reactions in nature [18]. Smell Agent Optimization (SAO) considers the relationship between odor agents and objects that vaporize odor molecules [19]. Ray Optimization (RO) [20] is proposed based on the idea of Snell’s light refraction law. Algorithms based on mathematical methods have also been extensively studied, for example, the Arithmetic Optimization Algorithm (AOA) [21] simulates the distributional properties of four basic operators. The Sine Cosine Algorithm (SCA) [22] is an algorithm proposed by the ideas of sine and cosine. Subtraction-Average-Based Optimizer (SABO) [23] proposes an individual updating the position of an individual by subtracting the average of the position idea.

**Figure 1:**
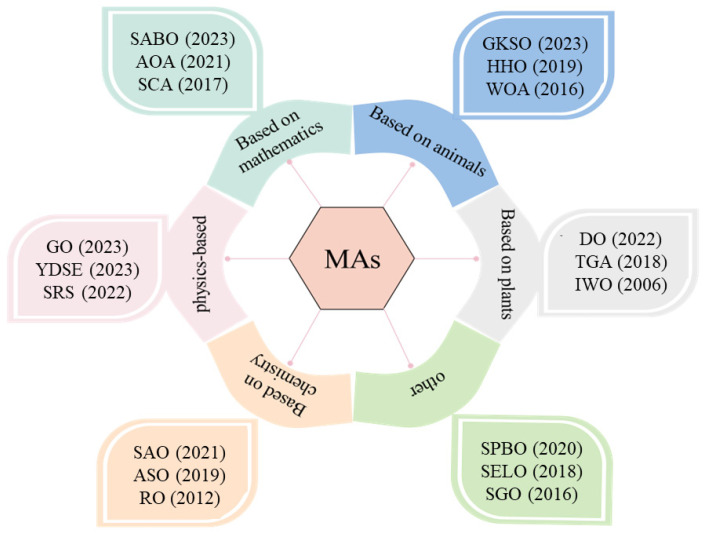
MAs classification figure.

Other types of algorithms include, but are not limited to, optimization algorithms based on human acquisition, e.g., Social Group Optimization (SGO) simulates the social behavior of humans in solving complex problems [24]. Social Evolution and Learning Optimization (SELO) simulates the social learning behavior of humans organized in families in social settings [25]. Student Psychology-Based Optimization (SPBO) simulates the process of students improving their level of proficiency in multiple ways during the learning process [26]. Further, the corresponding algorithms and publication dates are given, as shown in Figure 1.

To increase the broad application of MAs in various fields, researchers constantly update the design and performance of MAs and develop more efficient and accurate algorithms. Fatma A. Hashim [27] proposed a physics-based MA called Fick’s Law Algorithm (FLA) to optimize the process by constantly updating the position of molecules in different motion states under the concentration difference. It has been proven that FLA leads to optimal solutions with high robustness when confronted with real-world engineering optimization problems. However, some experimental studies have also found that FLA suffers from local convergence as well as degradation of convergence accuracy when faced with high-dimensional, high-complexity problems [28]. Therefore, we try to improve the FLA algorithm by introducing some efficient but non-redundant strategies and the proposed multi-strategy improved FLA algorithm (FLAS).

The proposed FLAS adopts many strategies to improve its performance and effect. Specifically, at the beginning of the exploration phase, the FLAS adds differential and Gaussian local mutation strategies to expand the search range in the later iteration. During the transition process phase, FLAS uses intersectional integrated learning strategies to enhance the ability to inquire about the overall situation and randomness. FLAS also adopts the Levy flight strategy in location updates and generates random steps with Levy distribution, which can carry out an extensive range of random searches in the search space. This randomness has an excellent global search ability and effectively controls the degree of the search and convergence rate. Finally, in the exploitation phase, FLAS also adds a global search mechanism for the migration phase of the seagull algorithm, aiming to speed up its convergence by avoiding molecular aggregation. Through the organic combination of these strategies, FLAS can effectively solve the problems of FLA in convergence accuracy and the convergence process and achieve better performance. The competitiveness and validity of FLAS are validated in 23 benchmark functions and CEC2020 tests, 7 engineering design problems, and solar PV parameter estimation applications. The results of FLAS are compared with those of other recent Mas, and are statistically analyzed using the Wilcoxon rank sum test.

The main contributions of this study are as follows:

(1) To overcome the shortcomings of the original algorithm FLA, a new optimization algorithm named FLAS is proposed by introducing the differential variational strategy, Gaussian local variational strategy, interleaving-based integrated learning strategy, and seagull updating strategy.

(2) Some classical and newly proposed algorithms are selected as comparison algorithms, and the optimization ability of FLAS is evaluated on 23 benchmark functions and the CEC2020 test set. The computational results of various performance metrics show that the proposed FLAS has the best overall performance in most of the tested functions.

(3) The FLAS is applied to seven engineering optimizations and the solar PV model parameter estimation, respectively. The results show that FLAS can stably provide the most reliable optimization design strategies for most practical problems.

The remainder of this paper is as follows: first, Section 2 analyzes, summarizes, and improves FLA. Then, in Section 3, the multi-strategy improved Fick algorithm is presented by adding five different strategies. Secondly, we test the performance of FLAS in Section 4. The results of FLAS on the CEC2020 test set are compared with other methods. In Section 5 and Section 6, FLAS is applied to seven practical engineering design problems and the solar PV parameter estimation application. Finally, this study is summarized and prospected.

## 2. An Overview of the FLA

**Figure 2:**
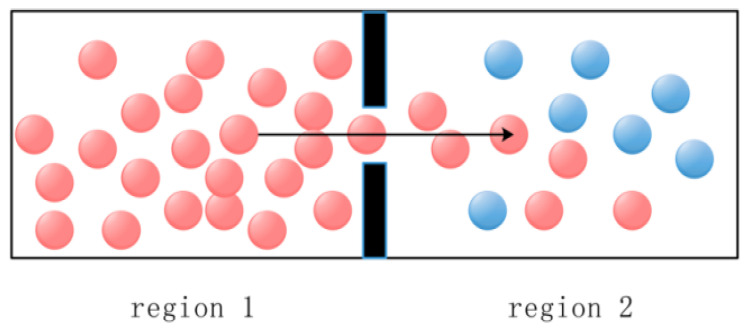
Molecule diffusion direction.

Fick’s law describes the fundamental principle of diffusion of substances in physics and chemistry. Therefore, Fick’s law algorithm simulates Fick’s law process of substance diffusion [27]. According to Fick’s law, the greater the concentration gradient, the faster the diffusion rate [29]. Therefore, Fick’s algorithm changes the concentration relationship between the two sides by adjusting the position of molecules in different regions to ensure a stable position of the molecules, thus realizing the optimization process. Figure 2 shows the schematic diagram of molecular movement in Fick’s law.

In the FLA algorithm, the optimization process first requires randomly initializing a set of candidate populations (***X***), whose matrix can be expressed as Equation (1).

(1) $$X=\left[\begin{array}{cccc} X_{1,1} & X_{1,2} & \cdots & X_{1,D}\\ X_{2,1} & X_{2,2} & \cdots & X_{2,D}\\ \vdots & \vdots & \vdots & \vdots \\ X_{N,1} & X_{N,2} & \cdots & X_{N,D} \end{array}\right],$$

where *N* is the population size, and *D* is the individual dimension. In addition, *X_i_*_,_*_j_* in the matrix represents the *j*th dimension of the *i*th molecule. The formula for random initialization is as follows:

(2) $$X_{i,:}=lb+rand(1,dim)\times (ub-lb),$$

where *ub* and *lb* represent the upper and lower bounds. The *rand* (1, *D*) is the random number uniformly generated in the search region. Divide *N* into two equal-sized subgroups, *N*_1_ and *N*_2_, and the fitness of the populations of *N*_1_ and *N*_2_ were calculated, respectively. The molecule constantly moved from high to low concentration, and *TF^t^* was a parameter of the iteration function. *TF^t^* is described by Equation (3).

(3) $$TF^{t}=\sinh{\left(t/T\right)}^{c_{1}}$$

where *t* represents the *t*th iteration and *T* represents the maximum number of iterations. sinh serves as a nonlinear transfer function that ensures an efficient transition from exploration to exploitation [27]. Further, the molecular position equation is updated by Equation (4).

(4) $$X_{i}^{t}=\left\{\begin{array}{l} \mathrm{DO}\;\;\;TF^{t}<0.9\\ \mathrm{EO}\;\;\;0.9\leq TF^{t}\leq 1\\ \mathrm{SSO}\;\;\;TF^{t}>1. \end{array}\right.$$

The FLA update process consists of three stages (diffusion operator (DO), equilibrium operator (EO), steady-state operator (SSO)). Through the three ways, FLA can find the best value of the system. We will specifically describe these three parts below.

### 2.1. Exploration Phase

**Figure 3:**
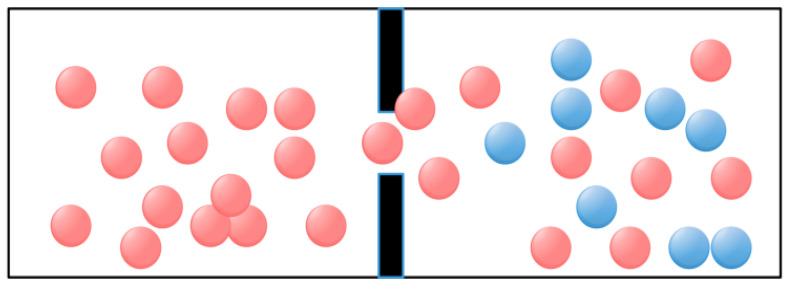
Molecule diffusion direction.

In the first stage, the diffusion of a molecule from high concentration to low concentration is called DO, as shown in Figure 3. When $TF^{t}<0.9$, due to the different concentration differences between the two regions *i* and *j*, the molecule will be transferred from one area to the other by the given parameter $T_{\mathrm{DO}}^{t}$, which can be provided by Equation (5):

(5) $$T_{\mathrm{DO}}^{t}=C_{5}\times TF^{t}-r$$

where *C*_5_ represents a fixed constant with a value of 2, and *r* means a random number with a value between 0 and 1.

From the parameter $T_{\mathrm{DO}}^{t}$, the flow direction of the molecule is determined by the following formula Equation (6).

(6) $$X_{p,i}^{t}=\left\{\begin{array}{c} \mathrm{from}\;i\;\mathrm{to}\;j\;\;\;T_{\mathrm{DO}}^{t}<rand\\ \mathrm{from}\;j\;\mathrm{to}\;i\;\;\;\;otherwise. \end{array}\right.$$

where *rand* is a random number with a value between 0 and 1. Consider that some molecules move from region *i* to region *j*. The formula for the number of molecules that move from region *i* to region *j* is as follows:

(7) $$NT_{ij}\approx N_{i}\times r_{1}\times (C_{4}-C_{3})+N_{i}\times C_{3}$$

where *C*_3_ and *C*_4_ represent the fixed constant with values of 0.1 and 0.2, respectively. *NT*_12_ and *Ntransfer* denote the number of molecules flowing at different stages, respectively. *r*_1_ is the random number in the interval [0, 1]. The specific formulae are as follows:

(8) $$NT_{12}\approx N_{1}\times r_{1}\times (C_{4}-C_{3})+N_{1}\times C_{3}$$

(9) $$Ntransfer\approx N_{2}\times r_{1}\times (C_{4}-C_{3})+N_{2}\times C_{3.}$$

The molecule *NT*_12_ will move to another, and its position will be updated mainly on the best-balanced molecule in the *j* region using Equation (10).

(10) $$X_{p,i}^{t+1}=X_{EO,j}^{t}+DF_{p,i}^{t}\times DOF\times r_{2}\times (J_{i,j}^{t}\times X_{EO,j}^{t}-X_{p,i}^{t+1}),$$

where *DOF* is the change of flow direction with time, $J_{i,j}^{t}$ is the diffusion flux, $X_{EO,i}^{t}$ denotes the balance position of region *i. r*_2_ is the random number in the interval [0, 1].

(11) $$DOF=\exp(-C_{2}(TF^{t}-r_{1})),$$

(12) $$J_{i,j}^{t}=-D\frac{dc_{i,j}^{t}}{dx_{i,j}^{t}},$$

(13) $$dc_{i,j}^{t}=X_{m,j}^{t}-X_{m,i}^{t},$$

(14) $$dx_{i,j}^{t}=\sqrt{{\left(X_{EO,j}^{t}\right)}^{2}-{\left(X_{p,i}^{t}\right)}^{2}+eps},$$

where *C*_2_ represents a fixed constant with a value of 2. $X_{m,j}^{t}$ and $X_{m,i}^{t}$ are the positions of the *j* and *i* regions, respectively and *eps* is a small value. *D* is the effective diffusion coefficient constant, $\frac{dc_{i,j}^{t}}{dx_{i,j}^{t}}$ is the concentration gradient. In addition, *NR*_1_ denotes molecules that remain in region *i*; the molecules in the *NR*_1_ are updated in their positions by Equation (15).

(15) $$NR_{1}\approx N_{1}-NT_{12}.$$

The positions of molecules in the formula are divided into three different strategies and position of region *i*; their positions do not change.

(16) $$X_{p,i}^{t+1}=\left\{\begin{array}{l} X_{EO,i}^{t},\;rand<0.8\\ X_{EO,i}^{t}+DOF\times (r_{3}\times (U-L)+L),\;rand<0.9\\ X_{p,i}^{t+1},\;otherwise \end{array}\right.$$

where *r*_3_ is the random number in the interval [0, 1]. *U* and *L* are the max and min limit.

For molecules in the *j* region, because the concentration in the *j* region is higher, the *j* region, boundary problem is treated with the following Equation (17):

(17) $$X_{p,j}^{t+1}=X_{EO,j}^{t}+DOF\times (r_{4}\times (U-L)+L),$$

where *r*_4_ is the random number in the interval [0, 1].

### 2.2. The Transition Phase from Exploration to Exploitation

**Figure 4:**
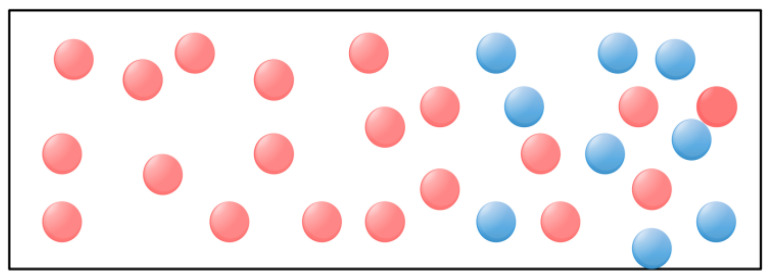
Molecule concentration equilibrium phase.

In the second phase, for the second stage where the concentration difference is almost zero, the molecule tries to reach an equilibrium state called *EO*, as shown in Figure 4.

This phase is considered a transition from the exploration phase to the exploitation phase. Molecules update their location by the following Equation (18).

(18) $$X_{p,g}^{t+1}=X_{EO,p}^{t}+Q_{EO,p}^{t}\times X_{p,g}^{t}+Q_{EO,p}^{t}\times (MS_{p,EO}^{t}\times X_{EO,g}^{t}-X_{p,g}^{t}),$$

where $X_{p,g}^{t}$ and $X_{EO,g}^{t}$ are the positions in group *p* and *g*, $MS_{p,EO}^{t}$ is the relative quantity of group *g*, calculated by the following Equation (25). $Q_{EO,g}^{t}$ is the diffusion rate factor of the group *g* region, and the calculation formula is as follows:

(19) $$Q_{EO,g}^{t}=R_{1}^{t}\times DF_{g}^{t}\times DRF_{EO,g}^{t},$$

where $DF_{g}^{t}$ is the direction factor equal to ±1, $R_{1}^{t}$ is a random number in the interval [0, 1], and $DRF_{EO,g}^{t}$ represents the diffusion rate.

(20) $$DRF_{EO,g}^{t}=\exp(-J_{p,EO}^{t}/TF^{t}),$$

(21) $$J_{p,EO}^{t}=-D\frac{dc_{g,EO}^{t}}{dx_{p,EO}^{t}},$$

(22) $$dc_{g,EO}^{t}=X_{g,EO}^{t}-X_{m,g}^{t},$$

(23) $$dx_{p,EO}^{t}=\sqrt{{\left(X_{g,EO}^{t}\right)}^{2}-{\left(X_{p,g}^{t}\right)}^{2}+eps},$$

(24) $$DF_{g}^{t}=\pm 1\;direction\;factor,$$

(25) $$MS_{p,EO}^{t}=\exp(-\frac{FS_{g,EO}^{t}}{FS_{p,g}^{t}+eps})\;motion\;step,$$

(26) $$R_{1}^{t}=rand{\left[0,1\right]}_{d}\;\;\;d=1:D.$$

where $FS_{g,EO}^{t}$ is the optimum of group *g* at time *t*, and $FS_{i,g}^{t}$ is the optimum of molecule *p* in group *g* at time *t*.

### 2.3. Exploitation Phase

In the third phase, we move the barrier so that the molecule moves to the most stable position to achieve a more stable molecule distribution. In the SSO phase, the molecule updates its position by the following Equation (27).

(27) $$X_{p,g}^{t+1}=X_{ss}^{t}+Q_{g}^{t}\times X_{p,g}^{t}+Q_{g}^{t}\times (MS_{p,g}^{t}\times X_{ss}^{t}-X_{p,g}^{t}),$$

where $X_{ss}^{t}$ and $X_{p,g}^{t}$ are the position of the stable phase and *p* molecule, and $Q_{g}^{t}$ and $MS_{p,g}^{t}$ respectively represent the relative quantity of *g* region and the motion step, the calculation formula is as follows:

(28) $$Q_{g}^{t}=R_{1}^{t}\times DF_{g}^{t}\times DRF_{g}^{t},$$

(29) $$DRF_{g}^{t}=\exp(-J_{p,ss}^{t}/TF^{t}),$$

(30) $$MS_{p,g}^{t}=\exp(-\frac{FS_{ss}^{t}}{FS_{p,g}^{t}+eps}),$$

(31) $$J_{p,ss}^{t}=-D\frac{dc_{g,ss}^{t}}{dx_{p,ss}^{t}},$$

(32) $$dc_{g,ss}^{t}=X_{m,g}^{t}-X_{ss}^{t},$$

(33) $$dx_{p,ss}^{t}=\sqrt{{\left(X_{ss}^{t}\right)}^{2}-{\left(X_{p,g}^{t}\right)}^{2}+eps}.$$

From the above overview, the pseudo-code for FLA is obtained in Algorithm 1.

**Algorithm 1** FLA Algorithm

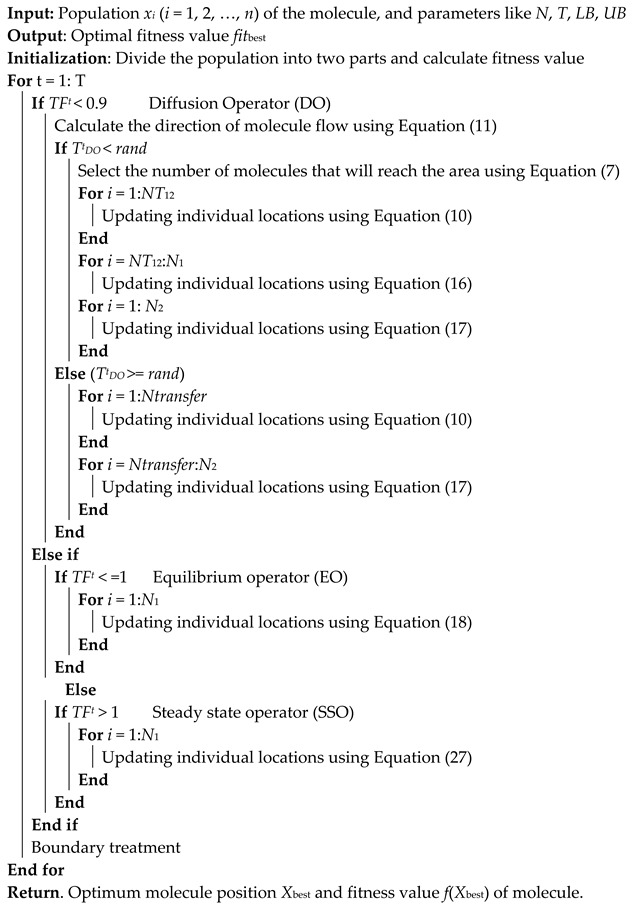



## 3. An Enhanced FLA (FLAS)

FLA can effectively mitigate the imbalance between exploration and utilization. However, it faces the same challenge as other MAs, being that it falls into local optimal solutions. Its main primitive is that the physical simulation search strategy of FLA leads to the inability of the molecules to get out of the local optimal solution. Therefore, to overcome this problem, this study proposes a multi-strategy improved FLA by introducing four effective search strategies, including differential variation, Gaussian local variation, Levy flight, and global search strategies.

### 3.1. Improvement Strategy

#### 3.1.1. Differential Variation Strategy

Differential variational strategy, as an effective strategy, has the advantages of diversity, efficient searchability, low computing cost, strong robustness, and ease of implementation and understanding [3]. Introducing random perturbations into the population helps algorithms maintain diversity in the search process and avoid the population falling into excessive convergence. At the same time, it can generate a new solution, often different from the parent solutions. Controlling the mutation operator makes more extensive exploration possible in the search space, which helps find potentially better solutions.

In the DO phase, FLA may have locally optimal solutions due to uneven diffusion of molecules when solving the optimization problem. Therefore, in order to better optimize the results, we try to introduce a differential variation strategy to improve the problem that the original algorithm tends to fall into local solutions. First, the weighted position difference between the two individuals is calculated. Further, the obtained result is then added to the position of a random individual to generate the variant individually. The specific formula is as follows:

(34) $$X1_{new}=X1+F_{0}\times (X1(randi(NT_{12}),:)-(X1(randi(NT_{12}),:),$$

where $X1_{new}$ represents the new position after updating, X1 represents the different individuals in the *t*th iteration, and $X1(rand(NT_{12}))$ represents the random individual position in the population *NT*_12_. In additional, *F* = 0.3 represents the scaling factor. The adaptive adjustment mutation operator *F*_0_ is described as follows:

(35) $$F_{0}=1+\frac{Fu^{2}}{NT_{12}^{2}}.$$

In addition, the differential variation strategy is more adaptable to the constraints of the boundary. By setting appropriate parameters, the amplitude and direction of variation operation can be controlled to ensure that the generated variation solution satisfies the constraint conditions of the problem.

#### 3.1.2. Gauss Local Variation Strategy

In fact, the development phase affects the convergence accuracy of the algorithm. To facilitate the low accuracy problem of FLA, we introduce a variational strategy in the development phase to improve the convergence performance. As a result, the Gaussian local variation can effectively help the algorithm further search for the optimal solution, expanding the search range of FLA in the later iteration stage [11]. The Gaussian local variation is specifically calculated by Equation (36):

(36) $$X1_{new}(i,j)=X1(i,j)+DOF\times ((ub(j)-lb(j))\times r_{3}+lb(j))\times normrnd(0,1).$$

where normrnd(0,1) represents a random number between 0 and 1. In the first *t* iteration, $X1_{new}(i,j)$ denotes the current individual optimum. *r*_3_ is the random number in the interval [0, 1].

#### 3.1.3. Integrated Learning Strategies Based on Intersections

In the DO and EO phases, the optimal individuals contribute to the bidirectional search of the whole population. However, the individual optimal values in FLA are not representative, and cannot lead to realizing the global search. Therefore, in order to obtain the most representative optimal individuals, we are inspired by the crossover operator and introduce a crossover-based comprehensive learning (CCL) strategy [12]. The CCL strategy can mutate better individuals through the crossover operator to guide the individual global search. The specific calculation formula is Equation (37):

(37) $$X1_{new}=r_{1}\times X1(i,j)+(1-r_{1})\times Xeo1+c_{1}\times (X1(i,j)-Xeo1),$$

where $c_{1}$ is a randomly generated number evenly distributed among [−1, 1], and both $r_{1}$ and $r_{2}$ are randomly numbers evenly distributed among [0, 1].

#### 3.1.4. Levy Flight Strategy

For better convergence and global optimization, the proposed FLAS employs Levy flight for position update of subgroup *N*_2_ [30]. After the FLA updates the position, a Levy flight is performed to update the individual position. Levy flight strategy is designed to simulate the stochastic and exploratory nature of Levy flights. It can perform a global search in the search space to achieve the desired result of jumping out of the local optimum. The specific calculation formula is described as Equation (38):

(38) $$X1_{new}=alfa\times levyrand(beta)\times X1,$$

where *levyrand* means the Levy distribution [30]. In addition, *alfa* means the levy scale parameter, and the value of *alfa* is 0.05 + 0.04 × *rand* and beta=2/3.

#### 3.1.5. Global Search Strategy of Gull Algorithm during Migration Phase

**Figure 5:**
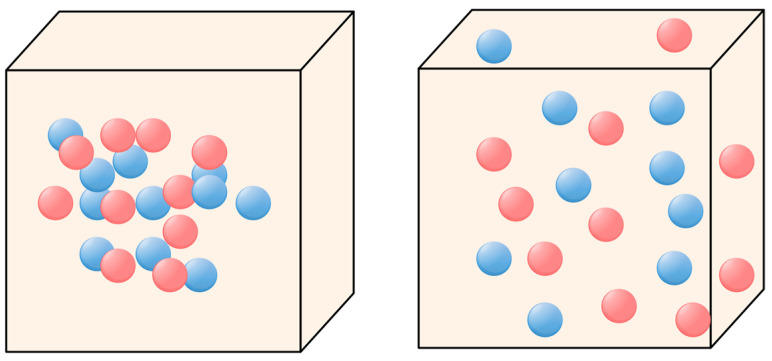
Collision avoidance of molecule.

In the stable phase (SSO), due to the local development of the algorithm, a large number of molecules may gather around the current environment, which restricts the development of the FLA, thus causing the FLA to be unable to break through its intrinsic limitations. At this time, the global search strategy of the seagull algorithm [31] is added to accelerate the convergence rate of FLA and avoid the collision between molecules in the process of motion [32]. ***D_alphs*** indicates that the new position of molecules has no conflict. The update process is shown in Figure 5, and the formula is as follows:

(39) $$D\_alphs=F_{c}\times X1(i,:)+A1\times (Xss-X1(i,:)),$$

where $F_{c}=2-\sin(I)\times \frac{2}{T}$ is the control factor, I=[0,0.8π], $A1=2\times F_{c}\times r_{1}-F_{c}$.

In the seagull optimization algorithm, the moving direction of each seagull individual, is calculated as the position. The moving distance is adjusted based on the fitness. The higher the fitness, the smaller the moving distance. Therefore, this strategy is employed to update the molecule positions during the stable phase. The specific position update formula is as follows

(40) $$Xe=D\_alphs\cdot e^{II}\times \cos(II\cdot 2\pi ),$$

where ***Xe*** is a new position that is reached by moving in the direction of the optimal position. $II=(F_{c}-1)\times rand+1$ represents a random number that balances global and local searches.

### 3.2. The Improved FLA Steps

The FLAS improved algorithm is established by introducing the differential variation strategy, Gaussian local variation strategy, interleave-based comprehensive learning strategy, Levy flight strategy, and the global search strategy in the migration phase of the Gull algorithm. The FLAS algorithm process is as follows:

When *TF^t^* < 0.9 and $T_{DO}^{t}<rand$, the position of *NT*_12_ obtained by Equation (10) in *N*_1_ population is updated by Equation (34).

(41) $$X1_{new}(i,\;)=X1_{new}(i,\;)+F_{0}\times (X1(Randi(NT_{12}),:)-(X1(Randi(NT_{12}),:)).$$

At the same time, for the remaining individuals (*N*_1_ − *NT*_12_) and the population individual position update, the strategies in Equations (36) and (37) are adopted. The formula is as follows:

(42) $$rand<0.8,\;X1_{new}(i,j)=Xeo1(j),$$

(43) $$rand<0.9,X1_{new}(i,j)=X1(i,j)+DOF\times ((ub(j)-lb(j))\times r_{3}+lb(j)\times normrnd(0,1),$$

(44) $$rand>0.9,X1_{new}(i,j)=r_{1}\times X1(i,j)+(1-r_{1})\times Xeo1+c_{1}\times X1(i,j)-Xeo1(j)).$$

In the *N*_2_ population, individual location updating adopts the strategies in Equations (36) and (38).

(45) $$X2_{new}(i,:)=Xeo2+DOF\times ((ub-lb)\times r_{4}+lb)\times normrnd(0,1),$$

(46) $$X2_{new}(i,:)=alfa\times levyrand(beta)\times X2_{new}(i,:).$$

when *T^t^_DO_* < *rand*, the position update of *Ntransfer* obtained with Equation (10) in *N*_2_ population is:

(47) $$X2_{new}(i,:)=Xeo1+DFg\times DOF\times rand(1,dim)\times (J\times Xeo1-X2(i,:)).$$

For the remaining individuals (*N*_2_ − *Ntransfer*), the same strategies in Equations (36) and (37) were adopted as the individual renewal mode of *N*_1_ population in *T^t^_DO_* < *rand* phase.

(48) $$rand<0.8,X2_{new}(i,j)=Xeo2(j),$$

(49) $$rand<0.9,X2_{new}(i,j)=X2(i,j)+DOF\times ((ub(j)-lb(j))\times r_{3}+lb(j)\times normrnd(0,1),$$

(50) $$rand>0.9,X2_{new}(i,j)=r_{1}\times X2(i,j)+(1-r_{1})\times Xeo2+c_{1}\times X2(i,j)-Xeo2(j)).$$

In this phase, the same strategies Equations (36) and (38) are used to update the positions of *N*_1_ population in the same phase as those of *N*_2_ population in *T^t^_DO_* < *rand* phase. The formula is as follows:

(51) $$X1_{new}(i,:)=Xeo1+DOF\times ((ub-lb)\times r_{4}+lb)\times normrnd(0,1),$$

(52) $$X1_{new}(i,:)=alfa\times levyrand(beta)\times X1_{new}(i,:).$$

When *TF^t^* ≤ 1, the EO phase will be entered, and the strategy Equation (36) will be adopted for both *N*_1_ population individual location renewal and *N*_2_ population individual location renewal. The formula is as follows:

(53) $$X1_{new}(i,:)=(Xeo1+Qeo\times X1(i,:)+Qeo\times (MS\times Xeo1-X1(i,:)\times normrnd(0,1),$$

(54) $$X1_{new}(i,:)=(Xeo2+Qeo\times X2(i,:)+Qeo\times (MS\times Xeo2-X1(i,:)\times normrnd(0,1).$$

When *TF^t^* > 1, entering the SSO phase, Equations (55) and (56) were adopted for individual location updates of the *N*_1_ population and *N*_2_ population, and the formula was as follows:

(55) $$X1_{new}(i,:)=Xss1+Qg\times Xe+Qg\times (MS\times Xss1-Xe),$$

(56) $$X2_{new}(i,:)=Xss2+Qg\times Xe+Qg\times (MS\times Xss2-Xe).$$

The basic steps of the FLAS:

Step 1. Specify both *N* and *T*, divide *N* into two equal small populations *N*_1_ and *N*_2_, randomly generate the initial positions of *N*_1_ and *N*_2_ individuals in the solution search space, and set the current iteration number *t* = 1.

Step 2. The fitness values of *N*_1_ and *N*_2_ individuals were calculated, and the optimal individual position was obtained.

Step 3. When *TF^t^* < 0.9, $T_{DO}^{t}\geq rand$ and *N*_1_ population enters the exploration phase (DO), and Equation (41) is used to update individual locations for *NT*_12_.

Step 4. When $T_{DO}^{t}\geq rand$, for the remaining population of individuals in *N*_1_, if rand < 0.8, the individual location is updated using Equation (43); if 0.8 < *rand* < 0.9, the individual location is updated using Equation (44); Others, using Equation (45) to update individual position.

Step 5. When $T_{DO}^{t}\geq rand$, in the population of individuals in *N*_2_, the individual locations were updated using Equation (46).

Step 6. In the case of $T_{DO}^{t}<rand$, in the *N*_2_ population, individual positions are updated using Equation (46) for *Ntransfer*. For the remaining individuals (*N*_2_ − *Ntransfer*), if *rand* < 0.8, the individual position is updated using Equation (46); if 0.8 < *rand* < 0.9, the individual location is updated using Equation (49); otherwise, the individual location is updated using Equation (50).

Step 7. When $T_{DO}^{t}\geq rand$, individual positions are updated using Equation (52) for *N*_1_ populations.

Step 8. When *TF^t^* ≤ 1, individual positions are updated using Equation (53) for *N*_1_ populations and individual positions are updated using Equation (54) for *N*_2_ populations.

Step 9. When *TF^t^* > 1, individual positions of population *N*_1_ are updated using Equation (55), and those in populations of *N*_2_ are updated using Equation (56).

Step 10. The boundary treatment of population location is carried out.

Step 11. Output the position and fitness values of the globally optimal individual.

The pseudo-code of FLAS is shown in Algorithm 2.

**Algorithm 2** FLAS Algorithm

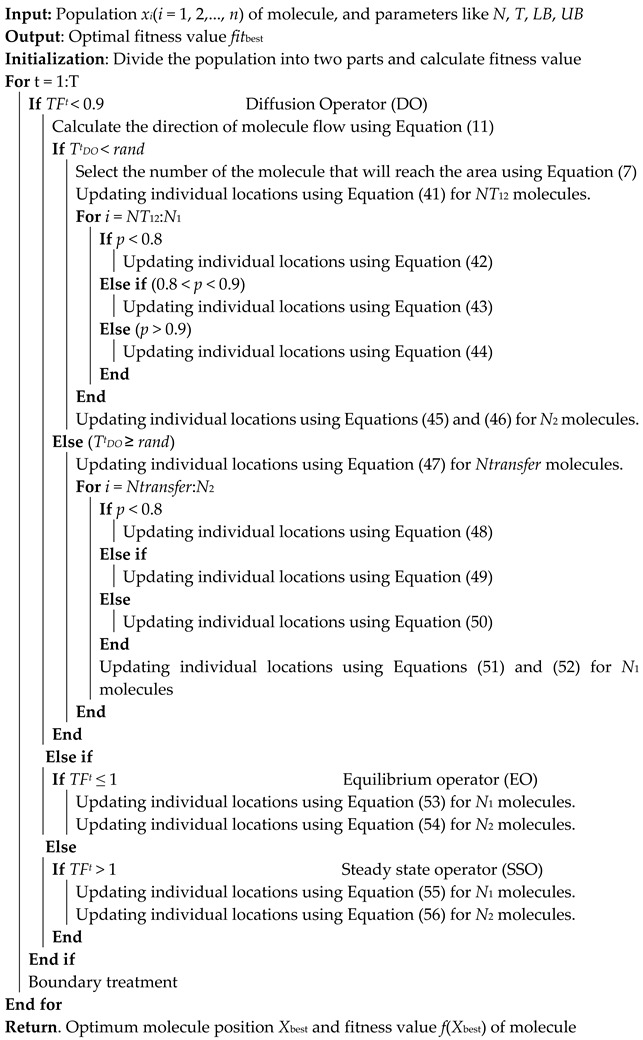



### 3.3. Time Complexity of the FLAS

MAs time complexity is influenced by both the dimensionality of variables *D*, population *N*, and iteration *T*. Determining the time complexity (*TC*) of an algorithm helps evaluate its operational efficiency. In the FLAS algorithm, first of all, the TC required during the initialization phase is *O*(*N* × *D*). FLAS then entered an iterative search for an updated solution. Entering the exploration phase. When the $T_{DO}^{t}<rand$, *TC* is *O*(*NT*_12_ × *D*) + *O*((*N*/2 − *NT*_12_) × *D*) + *O*(*N*/2 × *D*), when the $T_{DO}^{t}\geq rand$, TC is *O*(*Ntransfer* × *D*) + *O*((*N*/2 − *Ntransfer*) × *D*) + *O*(*N*/2 × *D*). The *TC* entering the EXE phase are *O*(*N*/2 × *D*) + *O*(*N*/2 × *D*). Finally, the *TC* for the exploitation phase is *O*(*N*/2 × *D*) + *O*(*N*/2 × *D*). The total *TC* of FLAS is calculated:

$$\begin{array}{l} O(FLAS)=O(N\times D)+O(T\times O(NT_{12}\times D)+O((N/2-NT_{12})\times D)\\ +\;O(Ntransfer\times D)+O((N/2-Ntransfer)\times D)\\ +\;O(6\times N/2\times D))=O(N\times D+(T\times 8\times N/2\times D))\\ =O(N\times D\times (1+4\times T)). \end{array}$$

**Figure 6:**
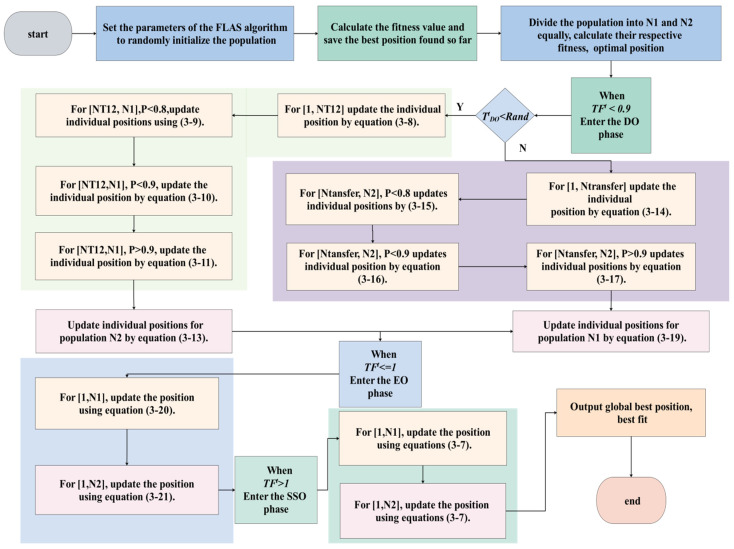
Algorithm flow chart of FLAS.

For a clearer description of the situation and the steps of FLAS to solve the optimization model, Figure 6 shows the algorithm solution flow chart.

## 4. Experimental Results

To verify the effectiveness of the FLAS method, the 23 benchmark functions and CEC2020 test sets are used to examine the optimization capability, and several algorithms have been selected for comparison. The selected comparison algorithms include the differential evolution algorithm (DE) [33], the improved molecule swarm optimization algorithm (PSO_ELPM) [34], the spectral optimization algorithm LSO [35] inspired by physics and mathematics, the arithmetic optimization algorithm AOA [36], the Harris Eagle algorithm (HHO) [7] inspired by animal nature, and the improved Golden Jackal optimization (IGJO) [37] and improved Grey Wolf algorithm (IGWO) [38]. The parameter values of the above 10 MAs are depicted in Table 1.

To accurately analyze the performance of FLAS, this article will use the following six performance metrics:

- $Best=\min\{best_{1},best_{2},\ldots ,best_{m}\}$;
- $Worst=\max\{best_{1},best_{2},\ldots ,best_{m}\}$;
- $Mean=\frac{1}{m}\sum_{i=1}^{m}best_{i}$;
- $std=\sqrt{\frac{1}{m-1}\sum_{i=1}^{m}{\left(best_{i}-Mean\right)}^{2}}$;

where *best_i_* represents the optimal result for the *i*th run, *m* implies the number of runs.

- Rank: All algorithms are ranked according to the quality of their performance indicators. The sequence number is the corresponding Rank. If the specific values of the comparison algorithms are equal, they are recorded as having the same Rank.
- Wilcoxon rank sum test: We estimate whether a noticeable disparity exists between the two algorithms. Calculate whether the two arrays of fitness values after m runs come from a continuous distribution with the same median. *p*-values derived by the Wilcoxon rank sum test for the other nine algorithms are shown at the α = 0.05 level. Bolded data show insignificant differences between the comparative algorithms and FLAS calculations. The “=” symbol indicates the number where there is no distinct difference between the results of other MAs and FLAS; the “+” symbol represents the number that outperformed FLAS, while the “−” symbol represents the number of functions that have inferior results compared to FLAS.

### 4.1. Parameter Analysis of Levy Scale Alfa

For the update strategy based on Levy flights, the parameter *alfa* affects the improvement of the algorithm performance with Levy flights. Due to the kinematic nature of each Levy flight, the random numbers it generates can only guide the overall convergence. The value of the parameter *alfa* affects the search range of the molecular neighborhood region. The larger the value of *alfa*, the larger the search range of the molecular neighborhood region and the greater the tendency of the algorithm to converge. If the value of *alfa* is too small, the search range of the molecular neighborhood region is small, and the Levy flight has less influence on the search process, which reduces the search capability and accuracy. Therefore, an appropriate value of *alfa* can improve the exploration ability of FLAS in individual molecular neighborhood locations.

In order to find an appropriate *alfa* value, the effects of different *alfa* values on the convergence performance of the FLAS algorithm are discussed. Ten test functions from the cec2020 test suite are selected for numerical experiments. Ten correlation parameters were obtained at intervals of 0.04 in the interval [0.01, 0.5] (0.01 + *rand* × 0.04, 0.05 + *rand* × 0.04, 0.1 + *rand* × 0.04, 0.15 + *rand* × 0.04, 0.2 + *rand* × 0.04, 0.25 + *rand* × 0.04, 0.3 + *rand* × 0.04, 0.35 + *rand* × 0.04, 0.4 + *rand* × 0.04 and 0.45 + *rand* × 0.04). For each given value of the ten parameters, the average values obtained using FALS over 20 independent runs of the test are shown in Table 2. The maximum iteration is 1000 and the population size is 30.

From the results in Table 2, it can be seen that the accuracy of the FLAS solution is higher when the value of α is taken as 0.05 + 0.04 × *rand*. The reason for this result is that smaller *alfa* allows FLAS to increase the distance connection between the population and the most available position, which enhances the local development ability of FLAS. Smaller values of *alfa* give better experimental results when faced with more complex mixing and combining functions. This result suggests that smaller *alfa* values increase the solution space search in the presence of more comprehensive functions and help the Levy flight strategy to better utilize its ability to jump out of local solutions. Thus, smaller values of *alfa* contribute to the local search ability of FLAS when dealing with complex functions. The last part of the table lists the rankings for different values of *alfa*. It can be noticed that FLAS performs well for all functions when *alfa* is 0.05 + 0.04 × *rand*.

### 4.2. Qualitative Analysis of FLAS

EXE are two important concepts in MAs. Exploration refers to looking for new solutions or improving existing solutions. Exploitation refers to the optimization and utilization of existing solutions in the hope of getting better results. To infer whether a group is currently more inclined to explore or exploit, it can be judged by counting the differences between individuals. If the differences between individuals are large, it indicates that the group is currently more inclined to explore; if the differences between individuals are small, it indicates that the group is currently more inclined to develop [39]. How the algorithm balances these two capabilities will be the key to determining optimal performance. Therefore, calculate the proportional formula of the two capabilities in the iterative process, as shown in Equation (58):

(57) $$Diversity_{j}=\frac{1}{N}\sum_{i=1}^{N}median(x_{ij})-x_{ij},$$

(58) $$Diversity=\frac{1}{D}\sum_{j=1}^{D}Diversity_{j}.$$

where median (*j*) represents the median value of the *J*-dimensional variable among all individuals in the population (*N*). After taking the median dimension distance of dimension j of each individual *i* in the *N*, the average *Diversity* is obtained for all individuals in turn. Then, the average value of *Diversity* in each dimension is calculated to obtain *Diversity*. The percentage of EXE of the population in each iteration formula are Equations (59) and (60).

(59) $$Exploration(\%)=\frac{Diversity}{Diversity_{\max}}\times 100$$

(60) $$Exploitation(\%)=\frac{\left|Diversity(t)-Diversity_{\max}\right|}{Diversity_{\max}}\times 100$$

**Figure 7:**
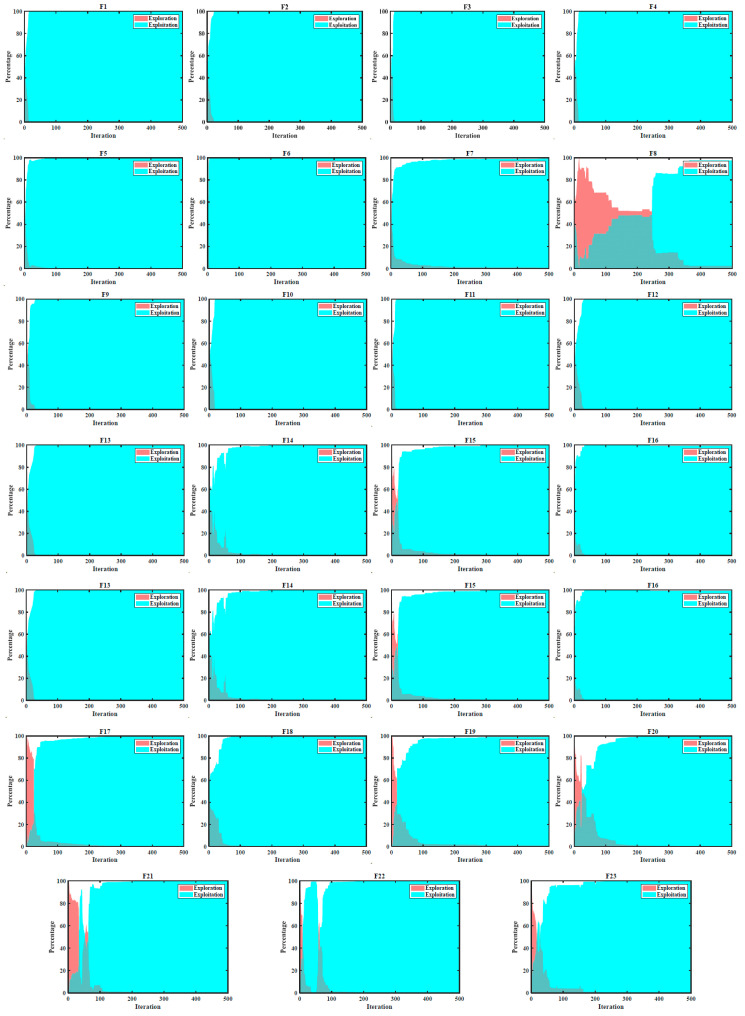
EXE diagram on 23 functions.

Firstly, the EXE ability of FLAS is tested on 23 benchmark functions, and the single-peak test function (F1–F7) is related to the capability of finding the optimal solution. The multimodal function (F8–F13) can test the MA’s capability to explore and escape local optima values because there are many local minima and the EXE ability of the fixed-dimensional multimodal function (F14–F23). The EXE diagram of FLAS is displayed in Figure 6. In the EXE diagram, Exploration is represented by the red area, and the blue area represents exploitation. The final exploitation should be close to 100% because the population gradually approaches optimum in the late phase of evolution, and the whole solution set is concentrated near optimum. In addition, the percentage of EXE should alternate as the iteration progresses: the blue area should go up and eventually approach 1; the red area is going to come down and eventually approach 0. Figure 7 shows that FLAS converges very fast on other functions except F8, F17, and F20, and the population soon approaches the optimal solution. According to the latest research [40], when EXE accounts for 10% and 90%, respectively, in the search process, the algorithm has the best performance. Therefore, FLAS meets this requirement; the Exploration of the population finally accounts for about 90%, and the exploitation accounts for about 10%.

**Figure 8:**
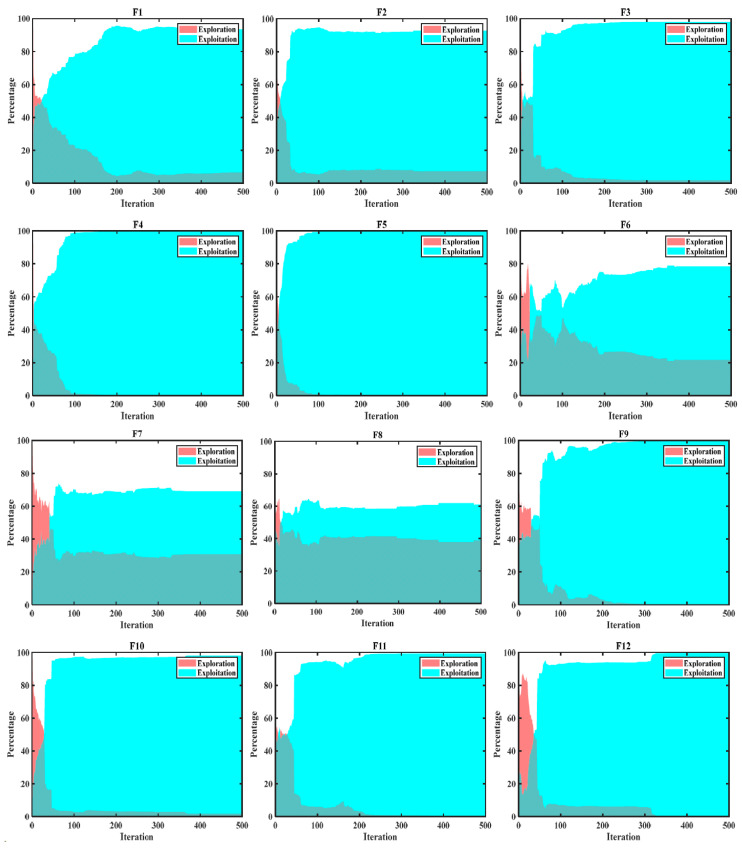
Exploration and development map on CEC2022.

To enhance the stability and reliability of the outcomes, give the ability to the EXE. FLAS continued testing on CEC2022. The EXE diagram on CEC2022 is shown in Figure 8, from which the FLAS demonstrates its ability to quickly find globally optimal solutions and flexibly switch between unimodal, fundamental, and combinatorial functions. When dealing with complex mixed functions, FLAS shows high exploration ability in the later iteration, and avoids the dilemma of local optimal solutions. FLAS has adopted a strategy to extend the time required to transition to the exploitation phase, effectively improving optimization capabilities.

### 4.3. Comparison of FLAS and Other MAs on 23 Benchmark Functions

**Figure 9:**
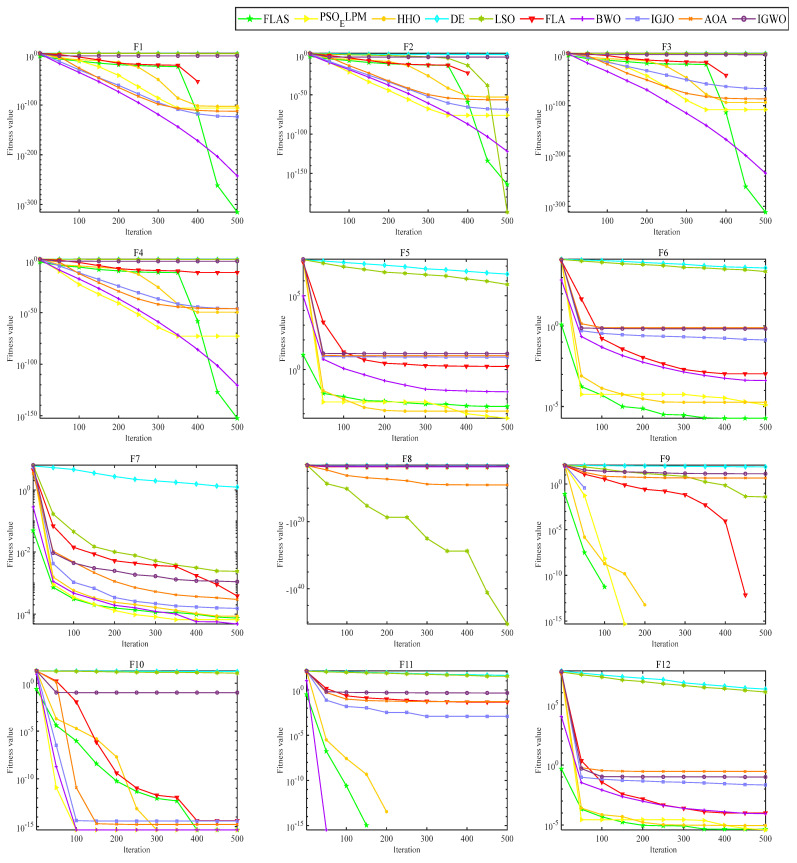

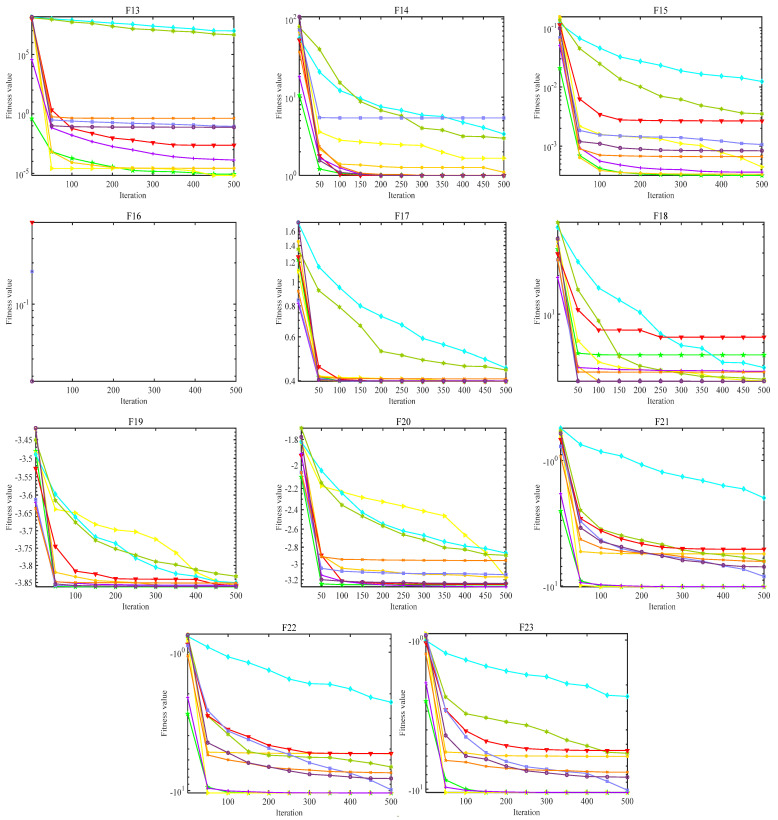
Convergence comparison diagram of FLAS and others.

**Figure 10:**
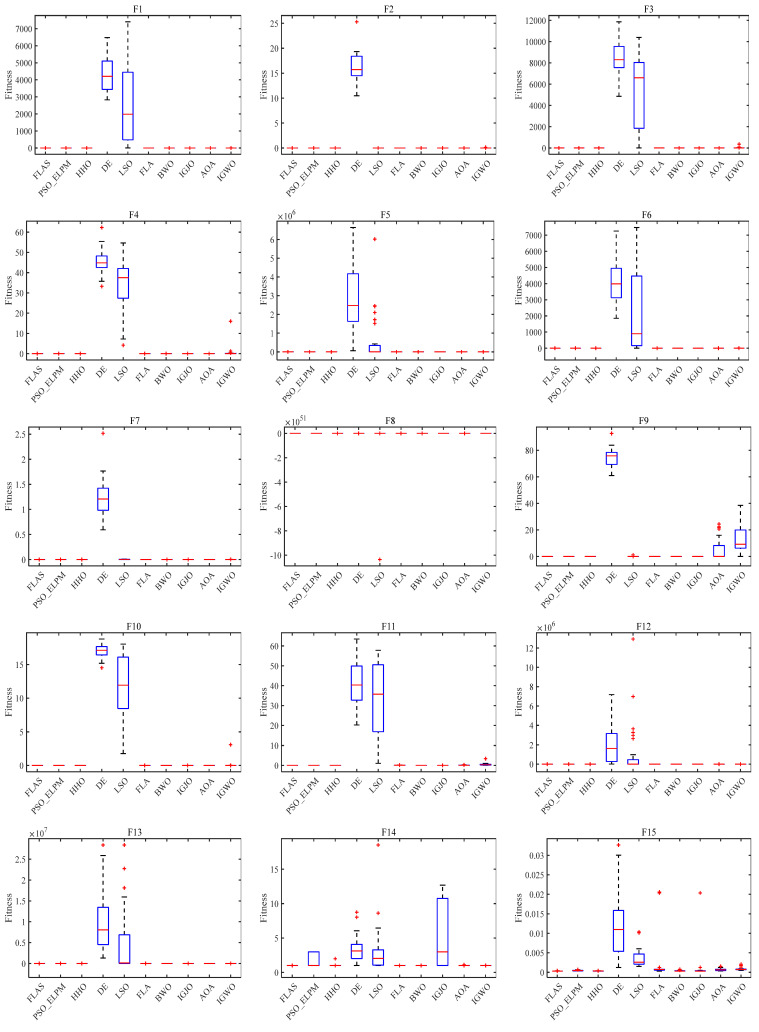

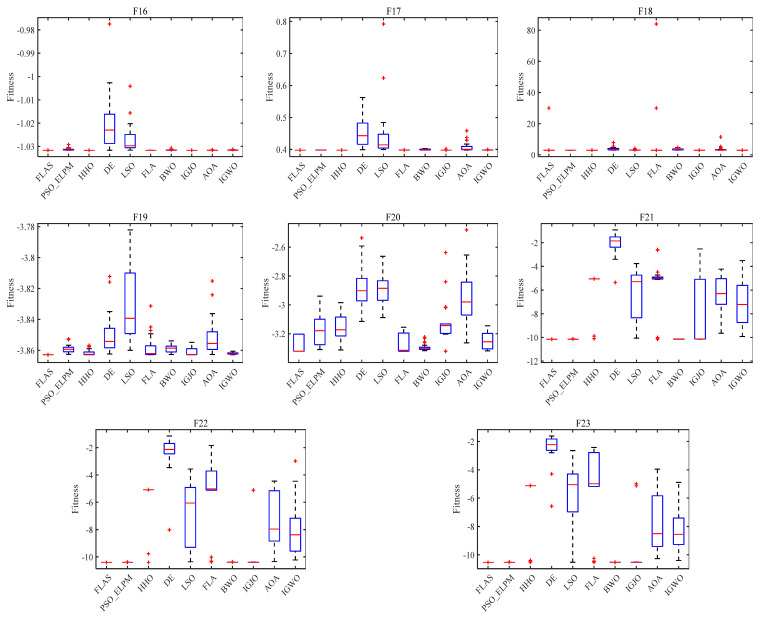
Box plot of FLAS and MAs on 23 benchmark functions.

In order to validate the effectiveness of the proposed algorithm for FLAS, comparison experiments with PSO_ELPM, HHO, IGWO, DE, LSO, BWO, AOA, and IGJO algorithms are conducted in 23 benchmark functions. All comparison algorithms are run 30 times. In addition, N is chosen to be 500 for all algorithms. The iterative plots and boxplots of FLAS and the other comparison algorithms for the 23 benchmark functions are given in Figure 9 and Figure 10, respectively. In addition, the results of the comparison experiments for the four metrics are given in Table 3. In order to statistically validate the effectiveness of the proposed algorithms, the statistical results of the Wilcoxon rank sum test for FLAS and other methods are given in Table 4.

As illustrated in Figure 9, the FLAS demonstrates noticeable superiority over other MAs, particularly from F1 to F7. For the multimodal test function, F8–F13, the FLAS outperforms other MAs in convergence speed and accuracy. For low-dimensional multi-peak test functions (F14–F23), FLAS is better than other MAs, particularly on F14, F15, and F20. Among the remaining functions, the optimum of 23 benchmark functions can be obtained accurately. For the F14–F23 function, we can see that FLAS can quickly transition between the early search and the late transition phase, converging near the optimal position at the beginning of the iteration. Then, FLAS progressively determines the optimal position and updates the answer to validate the previous observations. We can find that FLAS performs quite competitively across the three types of functions and maintains a consistent dominance in most of them. In addition, convincing results show that the FLAS algorithm is also able to balance both exploratory and developmental search. To infer information from Figure 10, the FLAS obtains lower and narrower boxes in most functions. The finding that in most cases, the distribution of the objectives of FLAS is centered on the other intelligent algorithms also illustrates the consistency and stability of FLAS. According to the comparison results between Table 3 and various algorithms, FLAS is generally the first. In addition, the proposed FLAS is ranked first for 15 of the test functions and is ranked second for the remaining 5 test functions. For the single-peak functions F1–F7, FLAS is ranked at least second in all of them, indicating that the proposed FLAS can find the single-peak optimal solution effectively. In addition, FLAS ranks first in all multi-peak functions, indicating that FLAS can effectively avoid the interference of localized solutions. Competitive performance is also demonstrated in complex problems such as fixed dimension. Considering the contingency of the test results, we further analyze the experimental results from the perspective of statistical tests. From Table 4, only two functions are better than the algorithm. FLAS is obviously superior to PSO_ELPM, HHO, DE, LSO, FLA, BWO, IGJO, AOA, and IGWO algorithms. Therefore, in the reference function, the FLAS converges significantly better. From the experimental results, it can be found that the proposed algorithm can effectively solve the single-peak as well as multi-peak optimization problems, and obtain better optimization results. But it can and will have a longer running time.

### 4.4. Comparison between FLAS and Other MAs on CEC2020

**Figure 11:**
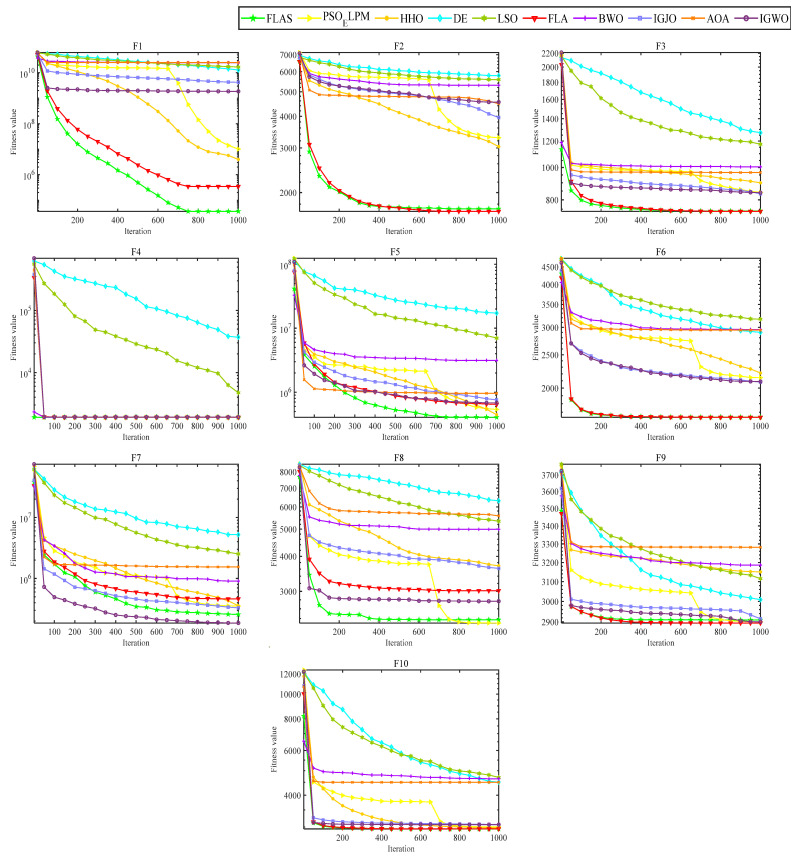
Convergence curves of different algorithms on CEC2020 test set.

**Figure 12:**
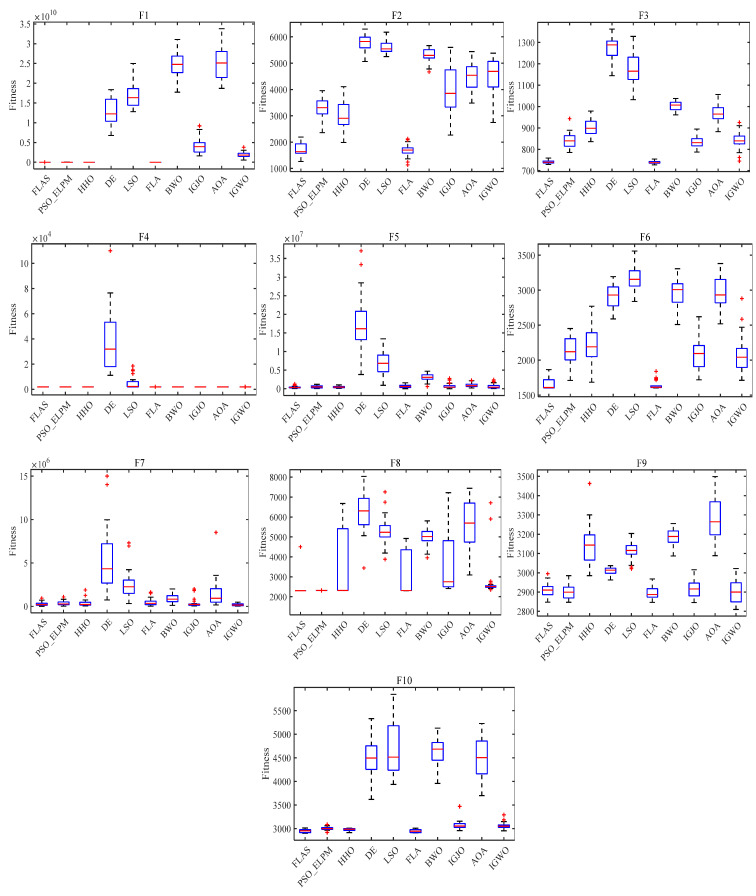
Box plot of different algorithms on CEC2020 test set.

**Figure 13:**
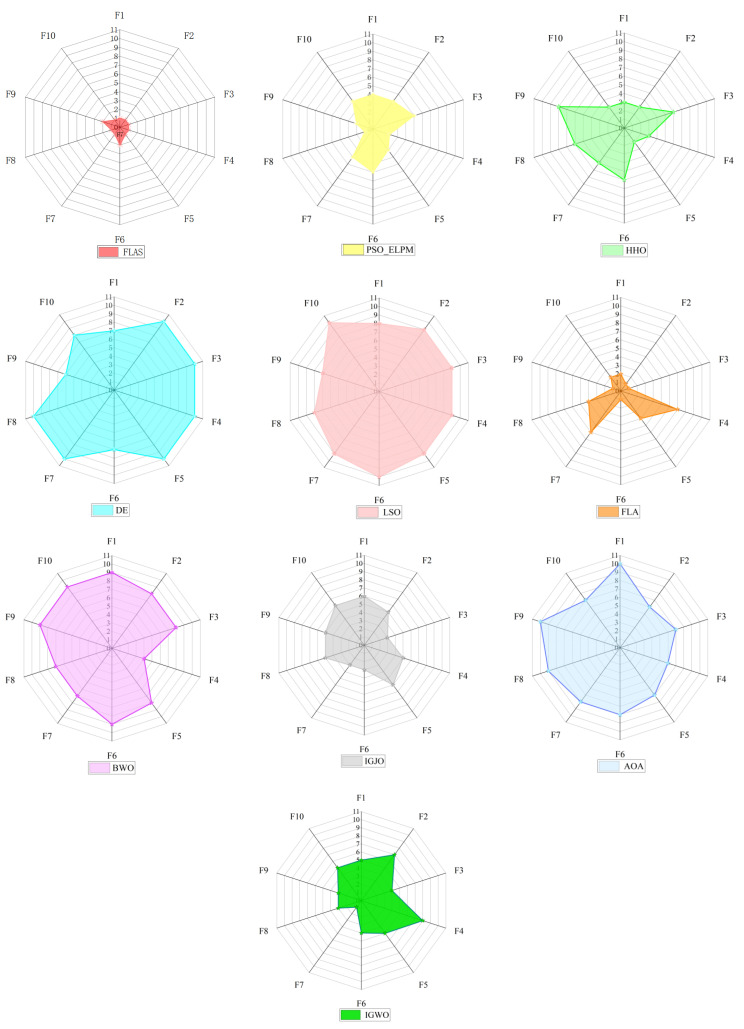
Radar map drawn with the rankings of different algorithms on the CEC2020 test set.

FLAS and other MAs are analyzed on the CEC2020 test set. The value has 20 dimensions and runs 30 times independently. As shown in Table 5, FLAS ranks first in F1, F4-F5, and F10. Among the ten algorithms, FLAS was in the top three on 90% of the tested functions. In Figure 11, FLAS converges the fastest of all functions except F6. Figure 12 shows a boxplot of MAs and data distribution for MAs. Figure 13 shows the radar plot of MAs. According to the data listed in Table 6, among the nine groups of comparison algorithms, only one function of the PSO_ELPM algorithm is superior to FLAS, and the others are inferior to FLAS.

### 4.5. Comparison of FLAS and DIRECT in CEC2020

**Figure 14:**
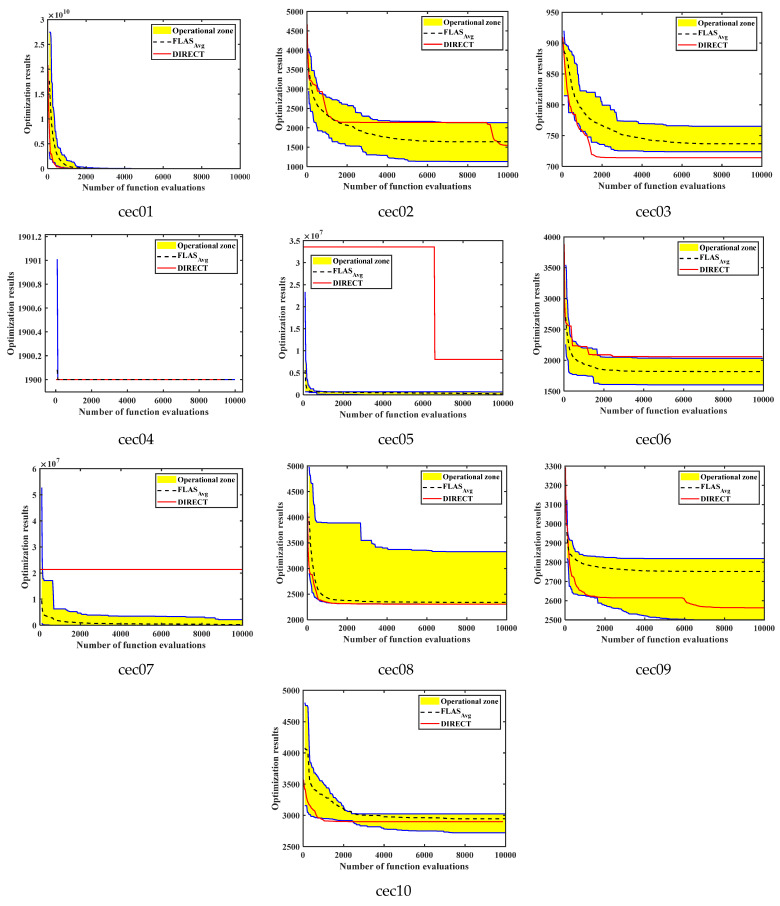
Operational zones built using 30 runs performed using the FLAS method and operational characteristics for the DIRECT method on ten CEC2020 functions. The upper and the lower boundaries of the zone are shown as dark blue curves.

In order to further validate the convergence and optimization performance of the proposed FLAS in the face of complex optimization problems, this section conducts experiments comparing FLAS with the well-known deterministic optimization method, DIRECT [41], in the CEC2020 test function. To ensure the validity of the experiments, both FLAS and DIRECT perform 10,000 function evaluation times [42]. Meanwhile, the population of FLAS is set to 30. for the CEC2020 test function, the dimension of all ten functions is set to 10. Figure 14 gives the operational zones built using 30 runs performed by the FLAS method and operational characteristics for the DIRECT method on ten CEC2020 functions. The upper and the lower boundaries of the zone are shown as dark blue curves [43].

As can be seen from the experimental result plots, the algorithmic performance of the proposed FLAS is competitive with the deterministic methods. FLAS demonstrates absolute optimization advantages in cec02, cec05, cec06, and cec07. The worst results of FLAS are comparable to or even better than those of DIRECT. For cec01, cec08, cec09, and cec10 test functions, the results of DIRECT optimize the average results of FLAS and are comparable to the best results of FLAS. In contrast, the optimal results for FLAS tend to be optimized better, indicating that FLAS has a higher upper bound. For cec04, both FLAS and DIRECT obtained optimal results. Only on cec03 did DIRECT obtain better results than FLAS. Thus, FLAS can also obtain better optimization results than deterministic methods.

**Figure 15:**
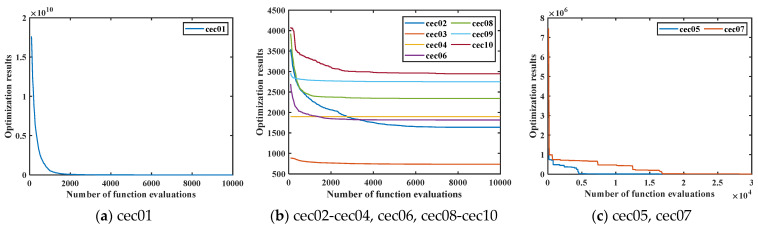
Convergence results for FLAS in CEC2020 test functions.

In addition, in order to effectively demonstrate the convergence situation of FLAS, we give plots of the convergence results of the ten test functions of CEC2020 for different numbers of function evaluations. Considering that the convergence speed of FLAS varies for different test functions, the number of function evaluations is set to 10,000 for the cec01–cec04, cec06, and cec08–cec10 test functions. For the cec05 and cec07 test functions, the number of function evaluations is set to 30,000. The graphs of the convergence of FLAS are given in Figure 15. In order to minimize the negative impact of the resultant magnitude on the presentation of the convergence plot results, only the convergence results for cec01 are given in Figure 15a.

From the results of the convergence plots, it can be found that FLAS guarantees effective convergence for different test functions.

## 5. Engineering Examples

In order to further evaluate the ability of FLAS to solve real-world applications, FLAS is compared with other well-performing MAs on many different engineering design problems. Two types of optimization problems are selected: (1) Box-constrained problems have only unique constraints on the upper and lower bounds of the variables, including gear transmission system design [44], and gas transmission compressor design [45]. (2) General-constrained problems have more complex constraints and include reducer design [46], three-bar truss design [47], piston rod optimization design [48], pressure vessel design [49], and stepped cone pulley problem [50]. We will conduct a comprehensive test and evaluation of FLAS through these specific engineering design questions to understand its performance and feasibility in practical applications [51]. This approach can help us better understand the advantages and limitations of FLAS and provide powerful solutions to optimization problems in the engineering field [52,53].

### 5.1. Reducer Design Problems

**Figure 16:**
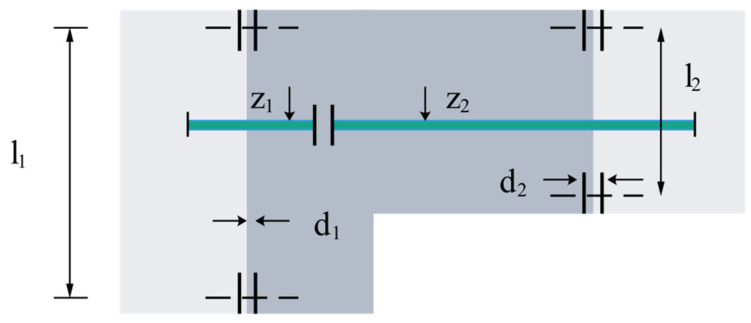
Reducer design problems.

Designing a reducer that meets specific requirements to achieve a speed ratio between a given input speed and an output speed achieves optimum [54]. In Figure 16, the reducer design problem has seven decision variables, namely, surface width ($x_{a_{1}}$), gear module ($x_{a_{2}}$), number of pinion teeth ($x_{a_{3}}$), bearing between the first axial length ($x_{a_{4}}$) and the second axis length ($x_{a_{5}}$), the first axis diameter ($x_{a_{6}}$), and the second axis diameter ($x_{a_{7}}$). From the figure, it can be understood that the problem requires optimization of the design weight under the constraints of bending stresses, surface stresses, transverse deflections, and axial stresses in the gear teeth. According to the definition of a gearbox, it is more complex than many practical applications because it has more constraints. The objective equation with respect to $x=[x_{a_{1}},x_{a_{2}},x_{a_{3}},x_{a_{4}},x_{a_{5}},x_{a_{6}},x_{a_{7}}]$ and 11 constraints are listed below.

(61) $$\begin{array}{l} \min\;f(x_{a})=0.7854x_{a_{1}}x_{a_{2}}^{2}(3.3333x_{a_{3}}^{2}+14.9334x_{a_{3}}-43.0934)\\ \;\;\;\;\;\;\;-1.508x_{a_{1}}(x_{a_{6}}^{2}+x_{a_{7}}^{2})+7.4777(x_{a_{6}}^{3}+x_{a_{7}}^{3})+0.7854(x_{a_{4}}x_{a_{6}}^{2}+x_{a_{5}}x_{a_{7}}^{2}), \end{array}$$

Variable values range from:

$$\begin{array}{l} 2.6\leq x_{a_{1}}\leq 3.6,\;0.7\leq x_{a_{2}}\leq 0.8,\;17\leq x_{a_{3}}\leq 28,\;7.3\leq x_{a_{4}}\leq 8.3,\\ 7.8\leq x_{a_{5}}\leq 8.3,\;2.9\leq x_{a_{6}}\leq 3.9,\;5.0\leq x_{a_{7}}\leq 5.5. \end{array}$$

The constraint conditions are:

$$\begin{array}{l} g_{1}(x_{a})=\frac{27}{x_{a_{1}}x_{a_{2}}^{2}x_{a_{3}}}-1\leq 0,\;g_{2}(x_{a})=\frac{397.5}{x_{a_{1}}x_{a_{2}}^{2}x_{a_{3}}^{2}}-1\leq 0,\;g_{3}(x_{a})=\frac{1.93x_{a_{4}}^{3}}{x_{a_{2}}x_{a_{3}}x_{a_{6}}^{4}}-1\leq 0,\;\\ g_{4}(x_{a})=\frac{1.93x_{a_{5}}^{3}}{x_{a_{2}}x_{a_{3}}x_{a_{7}}^{4}}-1\leq 0,\;g_{5}(x_{a})=\frac{\sqrt{{\left(745x_{a_{4}}/x_{a_{2}}x_{a_{3}}\right)}^{2}+16.9\times 10^{6}}}{110x_{a_{6}}^{3}}-1\leq 0,\;\\ g_{6}(x_{a})=\frac{\sqrt{{\left(745x_{a_{5}}/x_{a_{2}}x_{a_{3}}\right)}^{2}+157.5\times 10^{6}}}{85x_{a_{7}}^{3}}-1\leq 0,\;g_{7}(x_{a})=\frac{x_{a_{2}}x_{a_{3}}}{40}-1\leq 0,\;g_{8}(x_{a})=\frac{5x_{a_{2}}}{x_{a_{1}}}-1\leq 0,\;\\ g_{9}(x_{a})=\frac{x_{a_{1}}}{12x_{a_{2}}}-1\leq 0,\;g_{10}(x_{a})=\frac{1.56x_{a_{6}}+1.9}{x_{a_{4}}}-1\leq 0,\;g_{11}(x_{a})=\frac{1.1x_{a_{7}}+1.9}{x_{a_{5}}}-1\leq 0, \end{array}$$

By combining the FLAS with PSO_ELPM, HHO, DE, FLA, Beluga Whale Optimization algorithm (BWO) [55], Rat colony Optimizer (RSO) [56], Reptile search algorithm (RSA) [57], and the tunica swarm algorithm (TSA) [46], eight algorithms were compared. Table 7 shows the minimum total weight obtained using FLAS and other MAs. Moreover, the total weight obtained using FLAS is the smallest, which is 2997.0. The total weight obtained using RSO is larger, and its value is 8.76E+07. As shown in Table 8, the FLAS algorithm is the best in all metrics; their standard deviation is only 5.7526, which indicates that FLAS has a more accurate and stable result.

### 5.2. Three-Bar Truss Design

**Figure 17:**
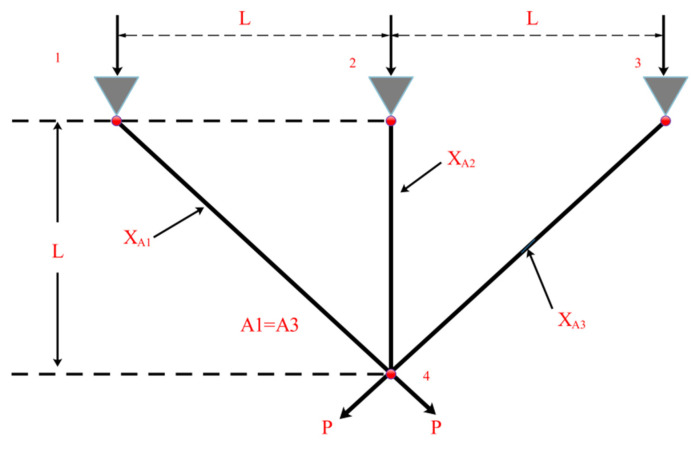
Design problems of three-bar truss design.

The objective of the three-bar truss design problem is to manipulate two parameters (*x_A_*_1_, *x_A_*_2_) to minimize the weight of the truss. The problem has three constraints: stress (σ), deflection, and buckling. A schematic diagram of the three-rod truss design problem is shown in Figure 17. Therefore, the mathematical model for the design of the three-bar truss is shown below:

Functions as follows:

(62) $$\min f(X)=(2\sqrt{2}x_{A1}+x_{A2})*l\;s.t.\left\{\begin{array}{l} g_{1}(X)=\frac{\sqrt{2}x_{1}+x_{2}}{\sqrt{2}x_{1}^{2}+2x_{1}x_{2}}P-\sigma \leq 0,\\ g_{2}(X)=\frac{x_{A2}}{\sqrt{2}x_{A1}^{2}+2x_{A1}x_{A2}}P-\sigma \leq 0,\\ g_{3}(X)=\frac{1}{\sqrt{2}x_{A2}+x_{A1}}P-\sigma \leq 0, \end{array}\right.$$

where $0\leq x_{A1},x_{A2}\leq 1$. In addition, l=100cm, $P=2\mathrm{KN}/{\mathrm{cm}}^{2}$, $\sigma =2\mathrm{KN}/{\mathrm{cm}}^{2}$.

Table 9 shows the HHO has the smallest function value, followed by the FLAS. However, according to Table 10, FLAS has obtained the best mean value. In addition to the slightly higher standard deviation, FLAS still has the first-solving effect.

### 5.3. Design Problems of Gear Group

**Figure 18:**
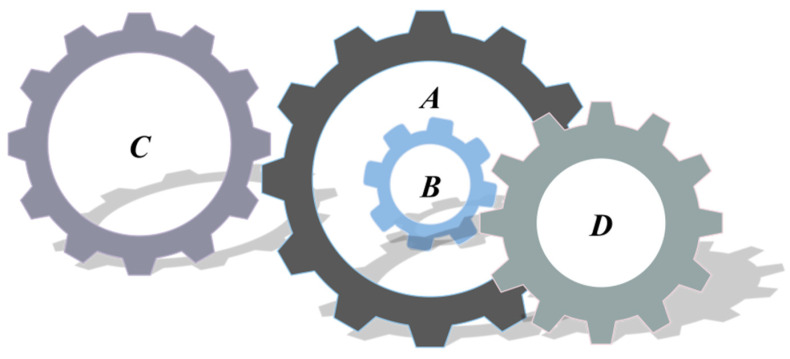
Design problem of gear group.

The number of teeth on the looking for the best to minimize the cost of gear ratios is the purpose of the gear set design problem [58], as shown in Figure 18. The problem is an integer unconstrained optimization problem with four design variables. These design variables denote the number of teeth of the gears and are denoted by $T_{A},T_{B},T_{C},T_{D}$, respectively [59,60].

Let $X=[x_{1},x_{2},x_{3},x_{4}]=[T_{A},T_{B},T_{C},T_{D}]$, and the following mathematical model is obtained:

(63) $$\min f(X)={\left(\frac{1}{6.931}-\frac{x_{1}x_{2}}{x_{3}x_{4}}\right)}^{2},$$

where $12\leq x_{1},x_{2},x_{3},x_{4}\leq 60$.

The proposed FLAS is compared with others on this problem. These include improved molecule swarm optimization (PSO_ELPM), Harris Eagle Optimizer (HHO), Differential Evolution Algorithm (DE), (SCA) [22] Fick algorithm (FLA), Beluga Whale Optimization Algorithm (BWO), Mouse Population Optimizer (RSO), Slime mold algorithm (SMA) [61], and improved Gray Wolf Algorithm (IGWO). In Table 11, except for DE, FLA, RSO, and SMA, the other algorithms all obtain minimum value, which indicates that MAs have the same effect on solving the extreme value of this problem. In Table 12, the reference index calculated using the FLAS algorithm is smaller. FLAS has the best solution effect and relatively stable results among these MAs.

### 5.4. Piston Rod Optimization Design

**Figure 19:**
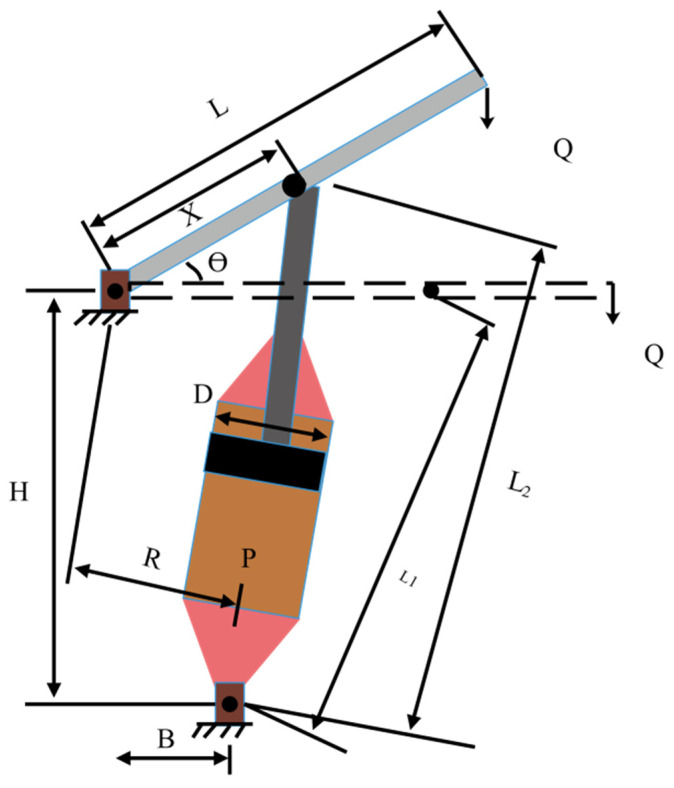
Schematic diagram of piston rod design.

The primary purpose of this engineering design is to guarantee that the oil volume is minimized during the lifting of the piston from 0° to 45° by positioning four different piston components (*H* (*w*_1_), *B* (*w*_2_), *D* (*w*_3_), and *X* (*w*_4_)). Figure 19 is a schematic of the piston rod [62], a mathematical model is developed as follows:

(64) $$\min\;f(w)=\frac{1}{4}\pi w_{3}^{2}(L_{2}-L_{1}),\;s.t.\;\left\{\begin{array}{l} g_{1}(w)=QL\cos\theta -R\times F\leq 0,g_{2}(w)=Q(L-w_{4})-M_{\max}\leq 0,\\ g_{3}(w)=1.2(L_{2}-L_{1})-L_{1}\leq 0,g_{4}(w)=\frac{w_{3}}{2}-w_{2}\leq 0, \end{array}\right.$$

where $R=\frac{\left|-w_{4}(w_{4}\sin\theta +w_{1})+w_{1}(w_{2}-w_{4}\cos\theta )\right|}{\sqrt{{\left(w_{4}-w_{2}\right)}^{2}+w_{1}^{2}}},\;F=\frac{\pi Pw_{3}^{2}}{4},\;L_{1}=\sqrt{{\left(w_{4}-w_{2}\right)}^{2}+w_{1}^{2}},$ and $L_{2}=\sqrt{{\left(w_{4}\sin\theta +w_{1}\right)}^{2}+{\left(w_{2}-w_{4}\cos\theta \right)}^{2}}$.

The variables *w*_1_, *w*_2_ and *w*_3_ fluctuate within [0.05, 500], and *w*_4_ belongs to [0.05, 120].

Table 13 and Table 14 compare FLAS with PSO_ELPM, HHO, DE, SCA, FLA, BWO, RSO, SMA, and IGWO, respectively. In Table 13, FLAS obtains the lowest cost. Table 14 shows that the results of FLAS are slightly better than those of other MAs. In addition, FLAS also obtained a small difference between the results obtained by 30 runs, which indicates that FLAS has better robustness while achieving optimal results.

### 5.5. Design of Gas Transmission Compressor

**Figure 20:**
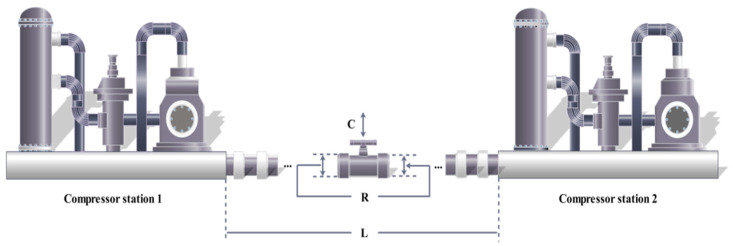
Design problems of gas transmission compressor.

In the minimization cost model, total costs need to be kept to a minimum, and *D*, *p_l_*, *p_s_*, *L*, *η*(*η* = *p_l_*/*p_s_*) are the correlation coefficient [3], as shown in Figure 20. Let $m=[m_{1},m_{2},m_{3}]=[L,\lambda ,D]$, and the following mathematical model is established:

(65) $$\begin{array}{c} \min f(m)=3.69\times 10^{4}m_{3}+7.72\times 10^{8}m_{1}^{-1}m_{2}^{0.219}-765.43\times 10^{6}\times m_{1}^{-1}\\ +\;8.61\times 10^{5}\times m_{1}^{\frac{1}{2}}{\left(m_{2}^{2}-1\right)}^{-\frac{1}{2}}m_{3}^{-\frac{2}{3}},\\ m_{1},m_{2},m_{3}>0,10\leq m_{1}\leq 55,1.1\leq m_{2}\leq 2,10\leq m_{3}\leq 40. \end{array}$$

The FLAS is compared with nine other MAs, namely FLAS with PSO_ELPM, HHO, DE, SCA, FLA, BWO, RSO, SMA, and IGWO. The solution results are shown in Table 15. By comparing the data of each algorithm in Table 16, FLAS is the best, which is 2,964,375.810043. Although the standard deviation of FLAS is not the smallest, the worst value and the mean value are also relatively small, which indicates that FLAS is more accurate than other comparison MAs.

### 5.6. Pressure Vessel Design Problems (PVD)

**Figure 21:**
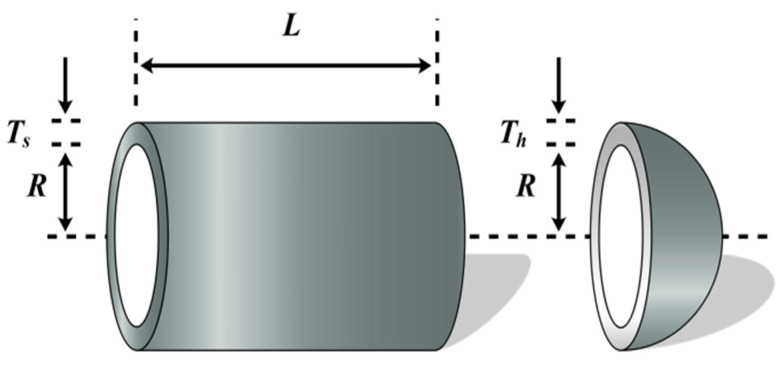
Pressure vessel design problems.

The ultimate requirement for pressure vessel design is to minimize the cost of fabrication, welding, and materials for the pressure vessel [63]. Figure 21 prompts us that a hemispherical head capped the cylindrical vessel at both ends. Four relevant design variables need to be considered for optimization, including shell thickness *T_s_*, head thickness *T_h_*, internal diameter *R*, and vessel cylindrical cross-section length *L*. Let $E=[e_{1},e_{2},e_{3},e_{4}]=[T_{s},T_{h},R,L]$. The mathematical optimization model for pressure vessel design is as follows:

(66) $$\min f(E)=0.6224e_{1}e_{3}e_{4}+1.7781e_{2}e_{3}^{2}+3.1661e_{1}^{2}e_{4}+19.84e_{1}^{2}e_{3}\;s.t.\left\{\begin{array}{l} g_{1}(E)=-e_{1}+0.0193e_{3}\leq 0,g_{2}(E)=-e_{2}+0.00954e_{3}\leq 0,\\ g_{3}(Ε)=-\pi e_{3}^{2}e_{4}-\frac{4}{3}\pi e_{3}^{3}+1,296,000\leq 0,g_{4}(E)=e_{4}-240\leq 0, \end{array}\right.$$

where $0\leq e_{1}\leq 99$, $0\leq e_{2}\leq 99$, $10\leq e_{3}\leq 200$, $10\leq e_{4}\leq 200.$

Therefore, FLAS is compared with PSO_ELPM, HHO, DE, SCA, FLA, BWO, RSO, SMA, and IGWO. From the data in Table 17 and Table 18, it is clear that the FLAS algorithm works best among the 10 MAs, which indicates that FLAS has good applicability in this problem.

### 5.7. Step Cone Pulley Problem

**Figure 22:**
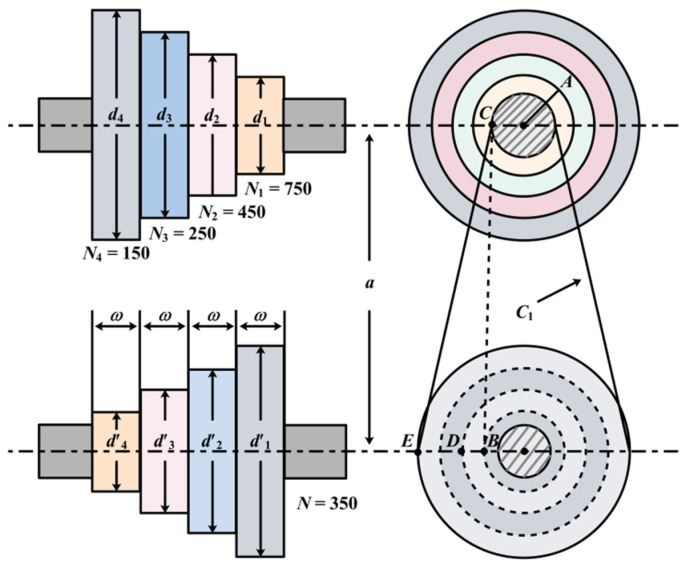
Problem of stepped cone pulley.

The goal of the step-cone pulley design problem is to design a four-step-cone pulley with minimum weight using five design variables, consisting of four design variables for the diameters of each step, with the fifth being the width of the pulley. Figure 22 displays the Step cone pulley problem. In this case, it is hypothesized that the tapered pulleys and the belt are of the same width. There are a total of 11 constraints, 3 of which are equality constraints and the rest are inequality constraints. The conditions are to make sure that the belt lengths, tension ratios, and power transmitted by the belt are the same in all steps. The design power of the stepped pulley is at least 0.75 hp (0.75 × 745.6998 W) with an input speed of 350 rpm and output speeds of 750, 450, 250, and 150 rpm, respectively [21]. The mathematical expression for the weight of the four-stage travel cone pulley that can be optimized is as follows:

(67) $$\min f(w)=\rho w_{5}[w_{1}^{2}\{1+{\left(\frac{M_{1}}{M}\right)}^{2}\}+w_{2}^{2}\{1+{\left(\frac{M_{2}}{M}\right)}^{2}\}+w_{3}^{2}\{1+{\left(\frac{M_{3}}{M}\right)}^{2}\}+w_{4}^{2}\{1+{\left(\frac{M_{4}}{M}\right)}^{2}\}],\;s.t.\left\{\begin{array}{l} h_{1}(w)=C_{b_{1}}-C_{b_{2}}=0,\;h_{2}(w)=C_{b_{1}}-C_{b_{3}}=0,\;h_{3}(w)=C_{b_{1}}-C_{b_{4}}=0,\\ Q_{i}(w)=R_{i}\geq 2\;(i=1,2,3,4),\\ Q_{i}(w)=P_{i}\geq (0.75\times 745.6998)\;(i=5,6,7,8), \end{array}\right.$$

where $\rho =7200\;{\mathrm{kg}/m}^{3},a=3\;m,\mu =0.35,s=1.75\;\mathrm{MPa},t=8\;\mathrm{mm},$ the mathematical expressions of *C_bi_*, *P_i_* and *R_i_* are, respectively:

$$\begin{array}{c} C_{b_{i}}=\frac{\pi d_{i}}{2}(1+\frac{N_{i}}{N})+\frac{{\left(\frac{N_{i}}{N}-1\right)}^{2}}{4a}+2a,\\ P_{i}=st\omega \;[1-\exp[-\mu \{\pi -2\sin^{-1}\{(\frac{N_{i}}{N}-1)\frac{d_{i}}{2a}\}\}]]\frac{\pi d_{i}N_{i}}{60},\\ R_{i}=\exp[\mu \{\pi -2\sin^{-1}\{(\frac{N_{i}}{N}-1)\frac{d_{i}}{2a}\}\}],\;(i=1,2,3,4) \end{array}$$

where, *t* = 8 mm, *s* = 1.75 MPa, *u* = 0.35, =7200 kg/m^3^, and *a* = 3 mm.

The FLAS is compared with PSO_ELPM, HHO, DE, SCA, FLA, BWO, RSO, SMA, and IGWO in solving the stepped cone pulley problem. Table 19 and Table 20 show that the optimal value of the FLAS algorithm is the closest to the real value among the 10 MAs, which fully indicates that FLAS works best.

## 6. Parameter Estimation of Solar Photovoltaic Model

The high-precision estimation of solar PV parameters is an urgent problem in power systems and the key to improving the output of power systems. In this section, parameters of the single diode model (SDM) in the photovoltaic (PV) model [64] are optimized by using to explore its effectiveness further. In the experiment, two sets of data [65] from the RTC France PV module and the Photowatt-PWP201 PV module were used to estimate the parameters of the SDM model of the RTC France PV module; the unknown parameters of the Photowatt-PWP201 PV module were estimated.

**Figure 23:**
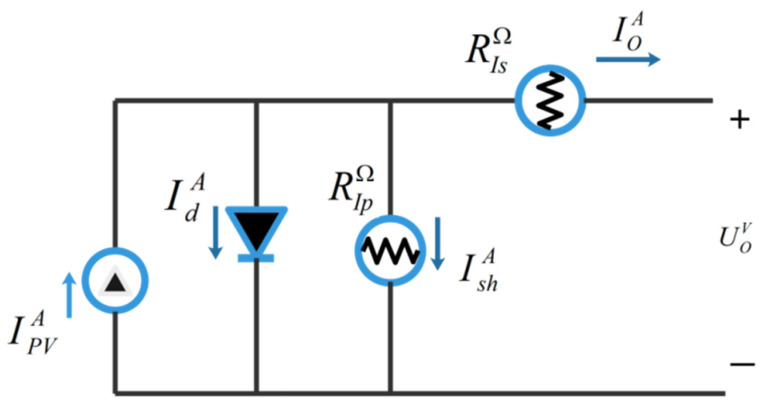
Schematic drawing of SDM.

Because of its simplicity, SDM has wide applications in simulating the performance of solar photovoltaic systems, and its structure is shown in Figure 23.

The relationship between the PV current, diode current and resistance current in the circuit and the output current is expressed as follows:

(68) $$I_{O}^{A}=I_{PV}^{A}-I_{d}^{A}-I_{sh}^{A},$$

(69) $$I_{d}^{A}=I_{Rs}^{A}\cdot [\exp(\frac{e(U_{O}^{V}+U_{Is}^{\Omega }\cdot I_{O}^{A})}{n\cdot l\cdot T_{K}})-1],$$

(70) $$I_{sh}^{A}=\frac{U_{O}^{V}+R_{Is}^{\Omega }\cdot I_{O}^{A}}{R_{Ip}^{\Omega }}.$$

where $I_{O}^{A}$ is the output current of SDM, $I_{PV}^{A}$ is the photovoltaic current, $I_{Rs}^{A}$ represents the reverse saturation current of SDM, $U_{O}^{V}$ is the output voltage, $R_{Is}^{\Omega }$ and $R_{Ip}^{\Omega }$ warm are the series resistance and parallel resistance of SDM, respectively, *e* is the electronic charge, usually 1.60217646 × 10-19C, *n* is the ideal factor of a single diode, *I* is the Boltzmann constant with a value of 1.3806503 × 10^−23^ J/K, and *T_k_* is the Kelvin temperature [66]. In Formula (68), since *I*, *e* and *T_k_* are all fixed constants, there are five parameters to be optimized in SDM: $I_{PV}^{A},\;I_{Rs}^{\Omega },\;I_{Ip}^{\Omega },\;I_{Is}^{A}$ and *n*, constituting a decision variable of ***X***_SDM_ = $\left[I_{PV}^{A},I_{Rs}^{\Omega },I_{Ip}^{\Omega },I_{Is}^{A},n\right]$ parameter optimization problem.

### PV Parameter Optimization Model and Experimental Setup

In a photovoltaic system, the output voltage $U_{O}^{V}$ and output current $I_{O}^{A}$ are the actual data measured in the experiment. The goal of the model is to find the values of unknown parameters [67] through MAs to minimize error. Jiao et al. [68] used the root-mean-square error to measure the deviation, and, based on this, they established a PV system parameter optimization model as follows:

(71) $$\min RMSE_{k}(X)=\sqrt{\frac{1}{N_{k}}\sum_{i=1}^{N_{k}}h_{ki}{\left(U_{Oi}^{V},I_{Oi}^{A},X\right)}^{2}},$$

where ***X*** represents the set of unknown parameters, *k* = 1 represents the label of the SDM module model, and *N_k_* represents the amount of data obtained for the *K*-th model. $h_{k}(U_{Oi}^{V},I_{Oi}^{A},X)$ represents the error when the *i*-th output voltage and current of the *k* type model are $U_{O}^{V}$, $I_{O}^{A}$, respectively.

(72) $$h_{k}(U_{Oi}^{V},I_{Oi}^{A},X)=I_{Oi,kleft}^{A}-I_{Oi,kright,}^{A}$$

where $I_{Oi,kleft}^{A}$ and $I_{Oi,kright}^{A}$ represent the left and right ends of the output current, respectively, FLAS is used to find the vector ***X*** that minimizes *RMSE*_k_(***X***). The absolute error (IAE) and relative error are used to evaluate the performance of the algorithm more accurately.

(73) $$IAE=|I_{O}^{A}-I_{Om}^{A}|$$

(74) $$\mathrm{RE}=\frac{I_{O}^{A}-I_{Om}^{A}}{I_{O}^{A}}$$

where $I_{O}^{A}$ is a real current value measured and $I_{Om}^{A}$ is a model current value calculated when the parameter is optimized.

In the experiment, the current and voltage values obtained with *N_k_* = 26 pairs of French RTC photovoltaic cells at temperatures of 1000 W/m^2^ and 33 °C were used as experimental data to estimate SDM and DDM model parameters. The current and voltage values of 36 polycrystalline silicon cells at 45 °C and low irradiance of 1000 W/m^2^ were connected as experimental data to estimate [69]. Table 21 lists some relevant parameters of SDM [70], *Lb* and *Ub*, on behalf of the upper and lower bounds. The parameters of all remaining algorithms are the same.

**Figure 24:**
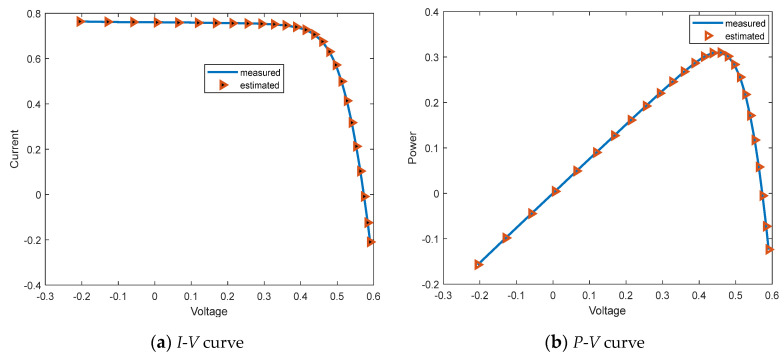

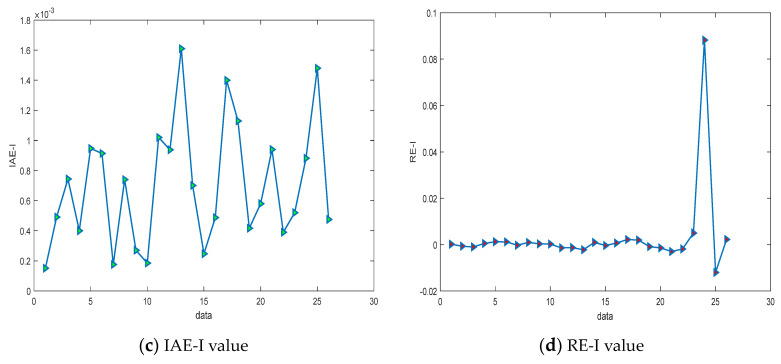
Schematic drawing of SDM.

Table 22 shows that FLAS performs well in performance evaluation compared to other MAs, significantly improving processing efficiency and achieving higher accuracy. This result undoubtedly strengthens the competitive advantage of FLAS in the field of intelligent computing. Table 22 shows the 26 groups of measured voltage *V*, current *I* and power *P* data of SDM, as well as the results of current *I*_m_, absolute current error *IAE*_I_ and absolute power error *IAE*_P_ estimated using FLAS; the absolute current error is all less than 1.61E-03. Combined with the curves in Figure 24a,b, the current data *I*_m_ and voltage data Pm calculated using FLAS are highly close to the actual data *I* and *P*. Figure 24c,d shows the *IAE* and *RE* of the simulated current; there is a high similarity between the experimental data and the estimated data. The FLAS is a method that can accurately estimate SDM parameters.

At the same time, using FLAS to seek SDM related Parameters, Table 23 shows the comparison results of five parameter values obtained using eight other MAs such as FLA, DMOA [69], IPSO [71], IGWO, ISSA [72], CSA [73], SCHO [74], and TSA to estimate SDM and *RMSE*. The *RMSE* value of FLAS is 1.09E-03. In summary, FLAS can find the solution faster, and the optimal solution is more accurate and stable. The results show that FLAS improves the output efficiency of the model.

## 7. Conclusions and Future Prospects

This paper proposes a multi-strategy augmented Fick’s law optimization algorithm to improve performance in facing high-dimensional and high-complexity problems, which combines the differential mutation strategy, Gaussian local mutation strategy, interweaving-based comprehensive learning strategy, and seagull update strategy. First, in the DO phase, FLAS improves the search diversity by adding differential and Gaussian local variation strategies, which further improves the aggregation efficiency and exploration capability in later iterations. In addition, the improved algorithm can effectively enhance search capability and stochasticity by introducing an integrated cross-based comprehensive learning strategy in the EO phase. Secondly, by introducing the Levy flight strategy in the position update, the Levy distribution can be effectively utilized to generate random steps to improve the search space’s overall randomization ability. Further, influenced by the idea of a seagull algorithm, FLAS introduces a migration strategy in the SSO stage to avoid the transition aggregation of molecules effectively. FLAS compares and analyzes other excellent improved algorithms, and the latest search algorithms on 23 benchmark functions and CEC2020. The results show that FLAS provides dominant results, especially when dealing with multimodal test functions. However, there is still room for further improvement in the ability of FLAS to face unimodal functions. In addition, the FLAS proposed in this paper is applied to seven real-world engineering optimization problems. The results show that the proposed algorithm has advantages in terms of computational power and convergence accuracy. Finally, FLAS is applied to the parameter estimation of solar PV models, and the experimental results demonstrate the applicability and potential of the proposed algorithm in engineering applications.

In future work, we will consider adding strategies at the initial population phase, such as Tent, Cubic chaos mapping, etc., and further improve the optimization capability of algorithm through different adaptive selection parameter values or combining them with other strategies. Enhanced optimized performance through more diverse test sets more challenging engineering applications for detailed testing. In addition, image feature selection [75,76], multi-objective problems [77], image segmentation [78,79], path planning [80,81,82], truss topology optimization [83], and shape optimization [84] can all be experimentally solved with FLAS.

## Figures and Tables

**Table 1:** Initial parameter Settings of all algorithms.

Algorithms	Parameter	Parameter Value
FLAS	Constant (*K*_1_, *K*_2_, *K*_3_, *K*_4_, *K*_5_, *D*)	*K*_1_ = 0.5; *K*_2_ = 2; *K*_3_ = 0.1; *K*_4_ = 0.2; *K*_5_ = 2; *D* = 0.01;
PSO_ELPM	Constant (*alpha*, *delta*, *u*)	*alpha* = 0.1; *delta* = 0.1; *u* = 0.0265;
HHO	Initial energy *E*_0_	*E*_0_ = 2 * *rand* () − 1;
DE	Control parameter *Cr*; Variation scaling factor *F*	*Cr* = 0.4; *F* = 0.5;
LSO	Constant (*Ps*; *Pe*; *Ph*; *B*)	*Ps* = 0.05; *Pe* = 0.6; *Ph* = 0.4; *B* = 0.05;
FLA	Constant (*G*_1_, *G*_2_, *G*_3_, *G*_4_, *G*_5_, *D*)	*G*_1_ = 0.5; *G*_2_ = 2; *G*_3_ = 0.1; *G*_4_ = 0.2; *G*_5_ = 2; *D* = 0.01;
BWO	Constant (*alpha*, *KD*)	*alpha* = 3/2; *KD* = 0.05;
IGJO	control parameter *E*1	*E*1 = 1.5 * (1 − (l/Maxiteration));
AOA	Constant (*B*_1_, *B*_2_, *B*_3_, *B*_4_, *u*, *l*)	*B*_1_ = 2; *B*_2_ = 6; *B*_3_ = 1; *B*_4_ = 2; *u* = 0.9; *l* = 0.1;
IGWO	Control parameter *a*	*a* = 2 − iter * ((2)/Maxiteration);

**Table 2:** Numerical results of FLAS for different values of the parameter *alfa*.

Functions	*alfa*, rank									
	0.01 + 0.04 × *rand*		0.05 + 0.04 × *rand*		0.1 + 0.04 × *rand*		0.15+0.04 × *rand*		0.2 + 0.04 × *rand*	
cec01	15,077.0282	6	12,709.1942	1	17,920.14697	10	15,364.27886	7	13,661.36227	3
cec02	1574.938322	5	1575.818157	6	1560.864712	3	1615.712912	9	1561.853461	4
cec03	726.6263789	2	727.6828071	8	727.4858505	5	726.7105188	3	726.041441	1
cec04	1900	1	1900	1	1900	1	1900	1	1900	1
cec05	61,152.90341	9	19,708.71303	4	27,162.80712	6	64,299.9035	10	34,461.35441	8
cec06	1828.744955	10	1752.75968	4	1759.339338	7	1755.905619	5	1746.317333	3
cec07	19,049.10676	10	8395.313019	3	12,561.99941	8	10,579.181	6	6714.90814	1
cec08	2303.174653	6	2299.072839	2	2298.039551	1	2299.304416	4	2357.749022	10
cec09	2741.012613	3	2740.518997	2	2765.743281	10	2737.713151	1	2759.224182	9
cec10	2934.147239	7	2930.806351	6	2935.532381	8	2925.942177	2	2912.618476	1
Total rank	59		37		59		48		41	
Functions	*alfa*, rank									
	0.25 + 0.04 × *rand*		0.3 + 0.04 × *rand*		0.35 + 0.04 × *rand*		0.4 + 0.04 × *rand*		0.45 + 0.04 × *rand*	
cec01	14,951.16004	5	15,854.54192	9	12,820.64997	2	14,083.00748	4	15,640.96252	8
cec02	1531.45172	2	1518.090145	1	1598.228861	8	1582.982073	7	1628.40018	10
cec03	729.7738857	10	727.6315489	7	729.228509	9	727.3692146	4	727.6055974	6
cec04	1900	1	1900	1	1900	1	1900	1	1900	1
cec05	12,017.4934	1	22,570.8507	5	18,248.68068	3	28,814.82339	7	15,926.23003	2
cec06	1781.727305	9	1725.543715	2	1764.464157	8	1758.572061	6	1716.517731	1
cec07	10,188.74183	5	11,290.39884	7	8892.825587	4	6925.282563	2	13,162.08088	9
cec08	2304.091653	8	2299.092661	3	2353.816844	9	2303.361191	7	2303.046363	5
cec09	2749.049576	6	2745.304211	4	2752.264893	8	2747.988309	5	2749.320034	7
cec10	2936.867379	10	2930.447954	5	2935.928651	9	2925.977361	3	2927.846478	4
Total rank	57		44		61		46		53	

**Table 3:** Experimental results of FLAS and MAs on 23 benchmark functions.

F	Index	FLAS	PSO-ELPM	HHO	DE	LSO	FLA	BWO	IGJO	AOA	IGWO
F1	Best	0	3.950E+03	6.085E-122	2.748E+03	8.675E-09	0	9.594E-253	2.464E-131	7.767E-141	1.831E-23
	Worst	2.619e-322	2.060E+04	9.563E-103	5.982E+03	7.578E+03	0	2.230E-241	3.054E-123	3.069E-102	5.133E-03
	Mean	9.881e-324	1.427E+04	4.099E-104	4.134E+03	3.170E+03	0	8.802E-243	2.500E-124	1.024E-103	1.805E-04
	Std	0.000E+00	3.540E+03	1.758E-103	8.371E+02	2.459E+03	0	0	6.603E-124	5.603E-103	9.357E-04
	Rank	2	10	5	9	8	1	3	4	6	7
F2	Best	0	3.037E+01	3.384E-62	7.800E+00	0.000E+00	0	1.053E-127	3.375E-71	3.268E-78	5.971E-20
	Worst	6.840E-159	2.308E+03	1.225E-51	2.355E+01	1.739E-01	0	5.219E-122	2.055E-66	7.706E-54	5.510E-03
	Mean	2.284E-160	4.568E+02	4.974E-53	1.669E+01	5.797E-03	0	5.908E-123	7.307E-68	2.574E-55	4.294E-04
	Std	1.249E-159	6.695E+02	2.237E-52	3.748E+00	3.175E-02	0	1.260E-122	3.744E-67	1.407E-54	1.282E-03
	Rank	2	10	6	9	8	1	3	4	5	7
F3	Best	0	6.289E+03	2.029E-108	4.306E+03	0.000E+00	0	9.010E-249	2.210E-76	1.463E-132	1.540E-05
	Worst	2.303E-298	2.567E+04	1.061E-84	1.567E+04	9.954E+03	0	2.576E-232	7.453E-65	5.338E-87	1.932E+02
	Mean	7.677E-300	1.509E+04	3.536E-86	8.041E+03	4.713E+03	0	9.617E-234	3.179E-66	1.779E-88	1.772E+01
	Std	0	5.170E+03	1.937E-85	2.476E+03	2.958E+03	0	0.000E+00	1.362E-65	9.745E-88	4.584E+01
	Rank	2	10	5	9	8	1	3	6	4	7
F4	Best	0	3.015E+01	1.020E-58	2.703E+01	3.305E+00	0	6.763E-126	8.298E-51	6.909E-64	8.449E-06
	Worst	9.548E-159	7.629E+01	5.870E-49	6.019E+01	5.202E+01	7.733E-09	4.473E-119	1.876E-46	3.971E-49	7.479E+00
	Mean	6.732E-160	6.482E+01	2.668E-50	4.398E+01	3.572E+01	6.070E-10	2.517E-120	1.379E-47	3.008E-50	4.429E-01
	Std	2.286E-159	9.157E+00	1.101E-49	6.845E+00	1.364E+01	1.643E-09	8.493E-120	3.731E-47	9.010E-50	1.415E+00
	Rank	1	10	3	9	8	6	2	5	4	7
F5	Best	1.437E-05	6.542E+06	4.616E-05	3.313E+05	8.766E+00	8.635E-03	4.710E-03	5.752E+00	8.266E+00	6.671E+00
	Worst	7.160E-02	6.327E+07	6.772E-03	4.984E+06	3.993E+06	1.389E+01	1.008E-01	8.701E+00	8.938E+00	3.549E+02
	Mean	5.775E-03	3.245E+07	1.207E-03	2.464E+06	3.909E+05	9.913E-01	3.377E-02	6.786E+00	8.658E+00	3.349E+01
	Std	1.444E-02	1.445E+07	1.397E-03	1.366E+06	8.554E+05	2.877E+00	2.293E-02	6.604E-01	1.285E-01	8.166E+01
	Rank	2	10	1	9	8	4	3	5	6	7
F6	Best	2.477E-10	7.672E+03	1.834E-07	1.539E+03	2.046E+00	5.829E-05	1.198E-04	6.650E-06	3.887E-01	1.723E-02
	Worst	2.130E-05	1.905E+04	5.891E-05	7.015E+03	8.885E+03	5.203E-03	5.261E-04	4.881E-01	1.114E+00	4.166E-01
	Mean	1.870E-06	1.402E+04	1.420E-05	4.016E+03	2.859E+03	1.066E-03	3.386E-04	1.156E-01	7.584E-01	8.227E-02
	Std	3.940E-06	3.378E+03	1.601E-05	1.281E+03	2.764E+03	1.206E-03	1.304E-04	1.521E-01	2.005E-01	9.068E-02
	Rank	1	10	2	9	8	4	3	6	7	5
F7	Best	2.12E-07	1.4075	5.83E-06	0.35449	0.00012896	4.02E-05	6.63E-06	6.90E-06	6.55E-05	1.20E-04
	Worst	1.62E-04	9.9939	2.12E-04	1.7041	0.011029	1.66E-03	3.09E-04	6.45E-04	1.06E-03	0.011526
	Mean	4.28E-05	5.3284	7.13E-05	1.0288	0.0023052	4.50E-04	8.81E-05	1.65E-04	3.83E-04	0.0013873
	Std	3.57E-05	2.6361	6.01E-05	0.39221	0.0025144	4.02E-04	6.80E-05	1.67E-04	2.60E-04	0.0020545
	Rank	1	10	2	9	8	6	3	4	5	7
F8	Best	−4.190E+03	−2.137E+03	−4.190E+03	−2.399E+03	−2.064E+48	−4.190E+03	−4.186E+03	−3.179E+03	−2.314E+08	−3.780E+03
	Worst	−3.479E+03	−1.806E+03	−2.648E+03	−1.669E+03	−8.041E+07	−4.071E+03	−3.451E+03	−1.744E+03	−4.084E+03	−2.179E+03
	Mean	−4.099E+03	−1.827E+03	−4.138E+03	−2.005E+03	−6.881E+46	−4.178E+03	−4.000E+03	−2.389E+03	−1.785E+07	−2.977E+03
	Std	1.695E+02	6.699E+01	2.815E+02	1.691E+02	3.768E+47	3.621E+01	2.435E+02	3.671E+02	5.104E+07	4.386E+02
	Rank	5	10	4	9	1	3	6	8	2	7
F9	Best	0	7.469E+01	0	4.064E+01	0	0	0	0	0	2.140E+00
	Worst	0	1.392E+02	0	9.267E+01	4.704E+01	9.950E-01	0	0	2.080E+01	4.135E+01
	Mean	0	1.142E+02	0	7.356E+01	2.100E+00	3.320E-02	0	0	3.460E+00	1.493E+01
	Std	0	1.379E+01	0	1.196E+01	8.890E+00	1.820E-01	0	0	7.180E+00	1.041E+01
	Rank	1	10	2	9	6	5	3	4	7	8
F10	Best	4.440E-16	1.846E+01	4.440E-16	1.173E+01	4.440E-16	4.000E-15	4.440E-16	4.440E-16	4.440E-16	2.040E-10
	Worst	4.440E-16	1.997E+01	4.440E-16	1.805E+01	1.853E+01	7.550E-15	4.440E-16	4.000E-15	4.000E-15	5.325E+00
	Mean	4.440E-16	1.973E+01	4.440E-16	1.671E+01	1.130E+01	4.230E-15	4.440E-16	3.520E-15	1.630E-15	1.842E-01
	Std	0	3.811E-01	0	1.312E+00	5.290E+00	9.010E-16	0	1.230E-15	1.700E-15	9.714E-01
	Rank	1	10	2	9	8	6	3	5	4	7
F11	Best	0	7.400E+01	0	1.549E+01	2.124E+00	0	0	0	0	1.240E-02
	Worst	0	1.818E+02	0	6.406E+01	8.576E+01	2.120E-01	0	0	5.339E-01	1.011E+00
	Mean	0	1.307E+02	0	3.862E+01	4.076E+01	4.902E-02	0	0	4.912E-02	3.595E-01
	Std	0	2.749E+01	0	9.445E+00	2.054E+01	5.351E-02	0	0	1.148E-01	2.891E-01
	Rank	1	10	2	8	9	5	3	4	6	7
F12	Best	1.190E-10	1.526E+07	1.040E-08	2.907E+04	4.620E-01	9.240E-06	8.260E-06	1.340E-06	6.810E-02	9.740E-04
	Worst	7.550E-05	1.437E+08	3.190E-05	1.130E+07	4.729E+06	2.530E-04	1.770E-04	7.870E-02	1.040E+00	5.965E+00
	Mean	5.090E-06	6.317E+07	7.580E-06	1.437E+06	5.600E+05	1.110E-04	8.630E-05	3.130E-02	2.710E-01	3.243E-01
	Std	1.380E-05	3.489E+07	8.600E-06	2.122E+06	1.400E+06	5.380E-05	4.280E-05	2.080E-02	1.890E-01	1.155E+00
	Rank	1	10	2	9	8	4	3	5	6	7
F13	Best	3.220E-07	2.630E+07	2.670E-08	7.094E+05	8.820E-01	6.370E-05	3.270E-05	1.500E-05	2.360E-01	8.880E-03
	Worst	8.940E-05	3.183E+08	2.920E-04	3.475E+07	2.373E+07	1.230E-02	3.310E-04	3.030E-01	8.270E-01	4.617E+00
	Mean	1.550E-05	1.349E+08	2.950E-05	8.968E+06	2.170E+06	2.030E-03	1.330E-04	9.420E-02	4.600E-01	2.836E-01
	Std	2.280E-05	6.868E+07	5.680E-05	7.319E+06	5.490E+06	3.800E-03	7.990E-05	9.110E-02	1.490E-01	8.684E-01
	Rank	1	10	2	9	8	4	3	5	7	6
F14	Best	9.980E-01	3.420E+00	9.980E-01	9.985E-01	9.980E-01	9.980E-01	9.980E-01	9.980E-01	9.980E-01	9.980E-01
	Worst	9.980E-01	4.169E+02	2.982E+00	9.161E+00	8.847E+00	9.980E-01	9.980E-01	1.267E+01	1.001E+00	1.040E+00
	Mean	9.980E-01	5.760E+01	1.230E+00	3.294E+00	3.278E+00	9.980E-01	9.980E-01	4.719E+00	9.982E-01	9.996E-01
	Std	1.725E-10	9.906E+01	5.005E-01	2.281E+00	2.423E+00	6.710E-10	5.007E-10	4.256E+00	6.394E-04	7.675E-03
	Rank	1	10	6	8	7	3	2	9	4	5
F15	Best	3.080E-04	1.012E-02	3.080E-04	1.828E-03	1.050E-03	3.150E-04	3.090E-04	3.080E-04	3.920E-04	4.270E-04
	Worst	3.990E-04	6.114E-01	1.340E-03	2.529E-02	2.603E-02	6.330E-02	5.020E-04	1.220E-03	2.090E-03	1.546E-03
	Mean	3.180E-04	9.282E-02	3.950E-04	1.141E-02	4.840E-03	3.440E-03	3.350E-04	4.620E-04	8.620E-04	7.579E-04
	Std	1.690E-05	1.052E-01	2.460E-04	6.806E-03	5.560E-03	1.190E-02	3.890E-05	2.770E-04	3.870E-04	2.237E-04
	Rank	1	10	3	9	8	7	2	4	6	5
F16	Best	−1.032E+00	−9.136E-01	−1.032E+00	−1.032E+00	−1.032E+00	−1.032E+00	−1.032E+00	−1.032E+00	−1.032E+00	−1.032E+00
	Worst	−1.032E+00	4.132E+00	−1.032E+00	−9.042E-01	−1.009E+00	−1.032E+00	−1.031E+00	−1.032E+00	−1.031E+00	−1.031E+00
	Mean	−1.032E+00	1.501E-01	−1.032E+00	−1.016E+00	−1.026E+00	−1.032E+00	−1.032E+00	−1.032E+00	−1.032E+00	−1.032E+00
	Std	7.278E-07	9.995E-01	1.288E-11	2.369E-02	5.881E-03	1.012E-06	8.137E-05	1.017E-07	1.230E-04	2.068E-04
	Rank	3	10	1	9	8	4	6	2	5	7
F17	Best	3.980E-01	4.110E-01	3.980E-01	3.981E-01	3.980E-01	3.980E-01	3.980E-01	3.980E-01	3.980E-01	3.980E-01
	Worst	3.980E-01	3.709E+00	3.980E-01	5.625E-01	5.304E-01	4.000E-01	4.110E-01	4.000E-01	4.840E-01	4.012E-01
	Mean	3.980E-01	1.482E+00	3.980E-01	4.542E-01	4.360E-01	3.980E-01	4.000E-01	3.980E-01	4.120E-01	3.983E-01
	Std	1.000E-05	8.000E-01	1.740E-06	5.073E-02	3.930E-02	4.620E-04	3.130E-03	3.360E-04	2.380E-02	7.667E-04
	Rank	2	10	1	9	8	4	6	3	7	5
F18	Best	3	3.155E+00	3	3.004E+00	3.000E+00	3.000E+00	3.000E+00	3.000E+00	3.000E+00	3.000E+00
	Worst	3	1.319E+02	3	9.748E+00	3.744E+00	3.000E+00	4.958E+00	3.000E+00	9.975E+00	3.030E+00
	Mean	3	4.081E+01	3	3.959E+00	3.086E+00	3.000E+00	3.396E+00	3.000E+00	3.414E+00	3.003E+00
	Std	5.161E-06	3.400E+01	2.599E-08	1.305E+00	1.498E-01	2.336E-05	5.152E-01	9.271E-07	1.331E+00	6.128E-03
	Rank	3	10	1	9	6	4	7	2	8	5
F19	Best	−3.860E+00	−3.782E+00	−3.860E+00	−3.861E+00	−3.860E+00	−3.860E+00	−3.860E+00	−3.860E+00	−3.860E+00	−3.860E+00
	Worst	−3.860E+00	−2.367E+00	−3.860E+00	−3.784E+00	−3.719E+00	−3.090E+00	−3.850E+00	−3.850E+00	−3.810E+00	−3.860E+00
	Mean	−3.860E+00	−3.393E+00	−3.860E+00	−3.841E+00	−3.820E+00	−3.840E+00	−3.860E+00	−3.860E+00	−3.850E+00	−3.862E+00
	Std	7.220E-07	3.343E-01	1.240E-03	1.901E-02	3.400E-02	1.410E-01	2.480E-03	3.560E-03	1.370E-02	7.008E-04
	Rank	1	10	3	7	9	8	5	4	6	2
F20	Best	-3.320E+00	-2.809E+00	-3.310E+00	-3.120E+00	-3.240E+00	-3.320E+00	-3.320E+00	-3.320E+00	-3.200E+00	-3.320E+00
	Worst	-3.200E+00	-9.615E-01	-2.830E+00	-2.306E+00	-2.377E+00	-3.170E+00	-3.200E+00	-3.020E+00	-2.470E+00	-3.156E+00
	Mean	-3.270E+00	-1.882E+00	-3.110E+00	-2.790E+00	-2.870E+00	-3.280E+00	-3.300E+00	-3.180E+00	-2.920E+00	-3.248E+00
	Std	6.030E-02	5.029E-01	1.300E-01	1.965E-01	1.800E-01	5.970E-02	2.380E-02	9.760E-02	1.800E-01	5.518E-02
	Rank	3	10	6	9	8	2	1	5	7	4
F21	Best	−1.015E+01	−1.421E+00	−1.013E+01	−4.805E+00	−1.015E+01	−1.012E+01	−1.015E+01	−1.015E+01	−9.951E+00	−9.835E+00
	Worst	−1.015E+01	−3.417E-01	−5.049E+00	−9.464E-01	−3.800E+00	−2.609E+00	−1.004E+01	−2.682E+00	−2.959E+00	−4.089E+00
	Mean	−1.015E+01	−5.840E-01	−5.386E+00	−2.025E+00	−7.079E+00	−6.273E+00	−1.015E+01	−8.647E+00	−6.812E+00	−6.363E+00
	Std	1.193E-03	2.493E-01	1.264E+00	9.376E-01	2.408E+00	2.592E+00	2.035E-02	2.570E+00	2.154E+00	1.860E+00
	Rank	1	10	8	9	4	7	2	3	5	6
F22	Best	−1.040E+01	−1.859E+00	−5.088E+00	−7.067E+00	−1.037E+01	−1.034E+01	−1.040E+01	−1.040E+01	−9.108E+00	−1.040E+01
	Worst	−1.040E+01	−3.932E-01	−5.081E+00	−1.151E+00	−2.554E+00	−1.818E+00	−1.034E+01	−5.108E+00	−2.510E+00	−2.494E+00
	Mean	−1.040E+01	−8.135E-01	−5.086E+00	−2.414E+00	−6.329E+00	−4.669E+00	−1.040E+01	−1.022E+01	−6.651E+00	−7.922E+00
	Std	6.842E-04	3.383E-01	1.585E-03	1.355E+00	2.577E+00	1.901E+00	1.163E-02	9.647E-01	1.875E+00	1.915E+00
	Rank	1	10	7	9	6	8	2	3	5	4
F23	Best	−1.054E+01	−1.704E+00	−1.053E+01	−4.233E+00	−1.053E+01	−1.053E+01	−1.054E+01	−1.053E+01	−1.023E+01	−1.023E+01
	Worst	−1.053E+01	−4.466E-01	−5.115E+00	−1.337E+00	−2.913E+00	−1.676E+00	−1.050E+01	−5.128E+00	−2.798E+00	−3.736E+00
	Mean	−1.054E+01	−9.062E-01	−5.307E+00	−2.211E+00	−6.205E+00	−5.302E+00	−1.053E+01	−9.990E+00	−7.092E+00	−8.115E+00
	Std	7.338E-04	3.164E-01	9.861E-01	6.607E-01	2.372E+00	2.517E+00	5.909E-03	1.640E+00	2.140E+00	1.657E+00
	Rank	1	10	7	9	6	8	2	3	5	4
Average rank		1.7	10.0	3.5	8.8	7.2	4.6	3.3	4.5	5.5	5.9
Final rank		1	10	3	9	8	5	2	4	6	7

**Table 4:** Wilcoxon rank sum test of FLAS and MAs on 23 benchmark functions.

F	PSO_ELPM	HHO	DE	LSO	FLA	BWO	IGJO	AOA	IGWO
F1	2.366E-12	2.366E-12	2.366E-12	2.366E-12	1.608E-01	2.366E-12	2.366E-12	2.366E-12	2.366E-12
F2	2.800E-11	2.800E-11	2.800E-11	8.496E-06	4.788E-08	2.800E-11	2.800E-11	2.800E-11	2.800E-11
F3	1.956E-11	1.956E-11	1.956E-11	1.624E-10	2.934E-05	1.956E-11	1.956E-11	1.956E-11	1.956E-11
F4	2.800E-11	2.800E-11	2.800E-11	2.800E-11	2.904E-02	2.800E-11	2.800E-11	2.800E-11	2.800E-11
F5	3.020E-11	4.119E-01	3.020E-11	3.020E-11	1.464E-10	6.518E-09	3.020E-11	3.020E-11	3.020E-11
F6	3.020E-11	5.186E-07	3.020E-11	3.020E-11	3.020E-11	3.020E-11	9.919E-11	3.020E-11	3.020E-11
F7	3.020E-11	7.013E-02	3.020E-11	3.338E-11	4.200E-10	1.767E-03	4.084E-05	1.094E-10	3.338E-11
F8	5.219E-12	4.290E-01	3.020E-11	3.020E-11	6.097E-03	1.041E-04	3.020E-11	1.695E-09	4.504E-11
F9	1.210E-12	NaN	1.210E-12	4.190E-02	3.340E-01	NaN	NaN	1.100E-02	1.210E-12
F10	6.870E-13	NaN	1.210E-12	1.660E-11	4.160E-14	NaN	1.970E-11	6.180E-04	1.210E-12
F11	1.212E-12	NaN	1.212E-12	1.212E-12	1.306E-07	NaN	NaN	6.617E-04	1.212E-12
F12	3.020E-11	9.880E-03	3.020E-11	3.020E-11	8.150E-11	1.460E-10	3.470E-10	3.020E-11	3.020E-11
F13	3.020E-11	7.620E-01	3.020E-11	3.020E-11	3.690E-11	3.470E-10	2.440E-09	3.020E-11	3.020E-11
F14	3.020E-11	8.766E-01	3.020E-11	3.338E-11	5.264E-04	3.183E-01	2.372E-10	3.338E-11	6.696E-11
F15	3.020E-11	8.120E-04	3.020E-11	3.020E-11	1.610E-10	1.370E-03	4.840E-02	3.690E-11	3.020E-11
F16	3.020E-11	4.491E-11	3.020E-11	3.020E-11	1.580E-01	4.200E-10	6.787E-02	1.996E-05	9.919E-11
F17	3.02E-11	4.71E-04	3.02E-11	3.02E-11	2.67E-09	4.08E-11	5.27E-05	4.20E-10	1.25E-07
F18	3.02E-11	3.02E-11	3.02E-11	3.02E-11	1.00E-03	3.02E-11	1.25E-07	3.02E-11	1.33E-10
F19	3.020E-11	3.340E-11	3.020E-11	3.020E-11	4.500E-11	3.020E-11	3.340E-11	3.020E-11	3.020E-11
F20	3.020E-11	2.030E-07	3.020E-11	1.210E-10	1.220E-02	5.200E-01	2.030E-07	3.020E-11	3.500E-03
F21	3.020E-11	3.020E-11	3.020E-11	3.338E-11	3.020E-11	1.850E-08	5.494E-11	3.020E-11	3.020E-11
F22	3.020E-11	3.020E-11	3.020E-11	3.020E-11	3.020E-11	1.311E-08	3.690E-11	3.020E-11	4.077E-11
F23	3.020E-11	3.020E-11	3.020E-11	3.020E-11	3.020E-11	1.011E-08	4.504E-11	3.020E-11	3.020E-11
+/=/−	0/0/23	0/8/15	0/0/23	0/1/22	1/2/20	0/5/18	1/3/20	0/1/22	0/0/23

**Table 5:** Experimental results of FLAS and comparison algorithm on CEC2020.

F	Index	FLAS	PSO_ELPM	HHO	DE	LSO	FLA	BWO	IGJO	AOA	IGWO
F1	Best	7.466E+06	8.032E+08	8.379E+07	1.046E+11	7.643E+10	3.711E+07	9.403E+10	1.604E+10	8.590E+10	3.678E+09
	Worst	3.003E+07	3.692E+09	3.827E+08	1.682E+11	1.271E+11	6.351E+07	1.101E+11	4.308E+10	1.184E+11	3.938E+10
	Mean	1.834E+07	1.714E+09	1.347E+08	1.291E+11	1.065E+11	4.689E+07	0	3.010E+10	1.040E+11	1.814E+10
	Std	5.277E+06	6.741E+08	5.266E+07	1.481E+10	1.379E+10	7.056E+06	4.320E+09	6.361E+09	8.753E+09	8.636E+09
	Rank	1	4	3	10	9	2	7	6	8	5
F2	Best	6.186E+03	6.341E+03	7.210E+03	1.475E+04	1.399E+04	5.894E+03	1.274E+04	7.608E+03	1.288E+04	6.712E+03
	Worst	9.313E+03	1.021E+04	1.018E+04	1.733E+04	1.679E+04	9.328E+03	1.497E+04	1.540E+04	1.508E+04	1.538E+04
	Mean	7.503E+03	8.223E+03	8.590E+03	1.651E+04	1.603E+04	7.749E+03	1.423E+04	1.096E+04	1.413E+04	1.282E+04
	Std	8.692E+02	9.685E+02	9.668E+02	5.490E+02	5.961E+02	7.884E+02	4.629E+02	2.475E+03	5.424E+02	2.784E+03
	Rank	1	3	4	10	9	2	8	5	7	6
F3	Best	9.779E+02	1.453E+03	1.779E+03	3.401E+03	2.072E+03	9.909E+02	2.012E+03	1.232E+03	1.899E+03	9.895E+02
	Worst	1.166E+03	1.768E+03	2.205E+03	4.324E+03	3.361E+03	1.245E+03	2.172E+03	1.778E+03	2.198E+03	1.672E+03
	Mean	1.069E+03	1.621E+03	1.964E+03	3.982E+03	2.788E+03	1.094E+03	2.113E+03	1.514E+03	2.097E+03	1.392E+03
	Std	5.121E+01	8.501E+01	9.869E+01	2.145E+02	4.015E+02	5.291E+01	3.710E+01	1.084E+02	7.650E+01	1.316E+02
	Rank	1	5	6	10	9	2	8	4	7	3
F4	Best	1.900E+03	1.900E+03	1.900E+03	6.834E+05	1.900E+03	1.900E+03	1.900E+03	1.900E+03	1.900E+03	1.903E+03
	Worst	1.900E+03	1.900E+03	1.900E+03	2.492E+06	3.795E+05	1.913E+03	1.900E+03	1.900E+03	1.900E+03	8.196E+03
	Mean	1.900E+03	1.900E+03	1.900E+03	1.411E+06	8.422E+04	1.906E+03	1.900E+03	1.900E+03	1.900E+03	2.245E+03
	Std	0.000E+00	1.411E-11	0.000E+00	4.032E+05	1.190E+05	5.373E+00	0.000E+00	0.000E+00	0.000E+00	1.188E+03
	Rank	1	6	2	10	9	7	3	4	5	8
F5	Best	7.665E+05	2.681E+06	1.736E+06	2.247E+08	2.480E+08	9.970E+05	1.527E+08	3.831E+06	2.262E+08	7.404E+06
	Worst	1.684E+07	3.189E+07	2.482E+07	1.087E+09	8.578E+08	2.618E+07	6.294E+08	2.282E+08	7.650E+08	1.622E+08
	Mean	7.848E+06	1.558E+07	9.098E+06	4.616E+08	4.782E+08	1.156E+07	3.620E+08	3.827E+07	4.674E+08	4.060E+07
	Std	4.851E+06	9.186E+06	5.653E+06	1.993E+08	1.466E+08	6.664E+06	1.106E+08	4.251E+07	1.494E+08	3.492E+07
	Rank	1	4	2	8	10	3	7	5	9	6
F6	Best	2.789E+03	3.233E+03	3.490E+03	6.714E+03	6.417E+03	2.271E+03	6.842E+03	3.057E+03	6.554E+03	2.715E+03
	Worst	4.409E+03	5.953E+03	5.792E+03	8.293E+03	8.958E+03	4.401E+03	9.539E+03	6.044E+03	9.949E+03	5.491E+03
	Mean	3.614E+03	4.217E+03	4.474E+03	7.470E+03	8.089E+03	3.434E+03	8.148E+03	4.140E+03	8.213E+03	4.485E+03
	Std	4.681E+02	6.873E+02	5.240E+02	4.342E+02	5.428E+02	4.185E+02	6.338E+02	5.844E+02	8.839E+02	6.094E+02
	Rank	2	4	5	7	8	1	9	3	10	6
F7	Best	9.050E+05	8.392E+06	2.495E+06	6.475E+07	1.340E+08	2.995E+06	8.500E+07	6.238E+05	2.980E+08	5.972E+06
	Worst	4.054E+07	8.249E+07	5.507E+07	5.131E+08	4.631E+08	5.691E+07	4.881E+08	1.121E+08	1.142E+09	7.568E+07
	Mean	1.386E+07	3.762E+07	2.044E+07	2.517E+08	2.705E+08	1.966E+07	2.490E+08	2.819E+07	6.584E+08	2.615E+07
	Std	1.145E+07	2.067E+07	1.614E+07	1.124E+08	9.122E+07	1.555E+07	9.274E+07	2.935E+07	2.329E+08	1.752E+07
	Rank	1	6	3	8	9	2	7	5	10	4
F8	Best	7.792E+03	7.168E+03	9.578E+03	1.662E+04	1.632E+04	8.083E+03	1.528E+04	9.071E+03	1.464E+04	3.095E+03
	Worst	1.059E+04	1.369E+04	1.438E+04	1.879E+04	1.840E+04	1.065E+04	1.746E+04	1.694E+04	1.723E+04	1.682E+04
	Mean	8.887E+03	1.131E+04	1.139E+04	1.791E+04	1.754E+04	9.282E+03	1.647E+04	1.278E+04	1.615E+04	1.229E+04
	Std	6.351E+02	1.282E+03	9.061E+02	5.764E+02	5.157E+02	6.825E+02	4.319E+02	2.531E+03	6.701E+02	4.254E+03
	Rank	1	3	4	10	9	2	8	6	7	5
F9	Best	3.177E+03	3.311E+03	3.887E+03	3.612E+03	3.990E+03	3.113E+03	4.100E+03	3.264E+03	4.598E+03	3.004E+03
	Worst	3.534E+03	3.643E+03	4.793E+03	3.989E+03	4.444E+03	3.415E+03	4.662E+03	3.650E+03	5.452E+03	4.126E+03
	Mean	3.328E+03	3.509E+03	4.212E+03	3.778E+03	4.246E+03	3.279E+03	4.426E+03	3.415E+03	4.992E+03	3.390E+03
	Std	8.446E+01	9.123E+01	2.386E+02	7.846E+01	9.993E+01	6.390E+01	1.175E+02	9.127E+01	2.591E+02	2.156E+02
	Rank	2	5	7	6	8	1	9	4	10	3
F10	Best	3.030E+03	3.213E+03	3.138E+03	1.578E+04	1.287E+04	3.030E+03	1.252E+04	4.187E+03	1.189E+04	3.747E+03
	Worst	3.216E+03	3.797E+03	3.307E+03	3.184E+04	2.565E+04	3.154E+03	1.463E+04	7.903E+03	1.635E+04	5.787E+03
	Mean	3.090E+03	3.469E+03	3.219E+03	2.487E+04	1.839E+04	3.095E+03	1.371E+04	5.577E+03	1.414E+04	4.383E+03
	Std	4.151E+01	1.197E+02	4.649E+01	4.421E+03	2.786E+03	3.541E+01	5.597E+02	8.881E+02	1.293E+03	4.824E+02
	Rank	1	4	3	10	9	2	7	6	8	5
Average rank		1.2	4.4	3.9	8.9	8.9	2.4	7.3	4.8	8.1	5.1
Final rank		1	4	3	10	9	2	7	5	8	6

**Table 6:** Wilcoxon rank sum test of 10 functions on CEC2020 using FLAS and other MAs.

F	Algorithms								
	PSO_ELPM	HHO	DE	LSO	FLA	BWO	IGJO	AOA	IGWO
F1	3.020E-11	3.020E-11	3.020E-11	3.020E-11	3.020E-11	3.020E-11	3.020E-11	3.020E-11	3.020E-11
F2	6.972E-03	2.839E-04	3.020E-11	3.020E-11	2.282E-01	3.020E-11	1.287E-09	3.020E-11	1.429E-08
F3	3.020E-11	3.020E-11	3.020E-11	3.020E-11	9.334E-02	3.020E-11	3.020E-11	3.020E-11	4.616E-10
F4	3.337E-01	NaN	1.212E-12	1.212E-12	2.213E-06	NaN	NaN	NaN	1.212E-12
F5	9.521E-04	5.395E-01	3.020E-11	3.020E-11	3.917E-02	3.020E-11	7.043E-07	3.020E-11	8.101E-10
F6	1.236E-03	1.157E-07	3.020E-11	3.020E-11	1.297E-01	3.020E-11	5.561E-04	3.020E-11	3.805E-07
F7	2.317E-06	1.055E-01	3.020E-11	3.020E-11	1.087E-01	3.020E-11	7.483E-02	3.020E-11	1.680E-03
F8	3.197E-09	6.696E-11	3.020E-11	3.020E-11	2.608E-02	3.020E-11	1.957E-10	3.020E-11	1.175E-04
F9	1.698E-08	3.020E-11	3.020E-11	3.020E-11	4.060E-02	3.020E-11	2.254E-04	3.020E-11	9.626E-02
F10	3.338E-11	1.613E-10	3.020E-11	3.020E-11	4.290E-01	3.020E-11	3.020E-11	3.020E-11	3.020E-11
+/=/−	1/4/5	0/3/7	0/0/10	0/0/10	0/5/5	0/1/9	0/3/7	0/1/9	0/3/7

**Table 7:** Optimal results for reducer design problems.

Algorithms	Optimal Variable							Optimal Weight
	*x_a_*_1_	*x_a_*_2_	*x_a_*_3_	*x_a_*_4_	*x_a_*_5_	*x_a_*_6_	*x_a_*_7_	
FLAS	3.5005	0.7000	17.0000	7.3000	7.8000	3.3512	5.2869	2997.0
PSO_ELPM	3.6000	0.7000	17.0000	8.3000	8.3000	3.4051	5.5000	3211.6
HHO	3.5202	0.7000	17.0000	7.8000	7.9000	3.3513	5.2913	3015.0
DE	3.5358	0.7000	17.0000	7.6000	8.0000	3.3515	5.3040	3029.4
FLA	3.5209	0.7000	17.0000	7.3000	8.1000	3.3635	5.2908	3082.1
BWO	2.8055	0.7000	17.0000	7.3000	7.8000	3.0775	5.0000	3017.7
RSO	3.5850	0.7000	17.0000	7.3000	8.3000	3.4128	5.5000	8.7600E+07
RSA	3.5076	0.7000	17.0000	7.3000	7.8000	3.3530	5.3106	3199.4
TSA	3.6000	0.7000	17.0000	7.5000	8.1000	3.3719	5.2884	3015.3

**Table 8:** Statistical results of reducer design problems.

Algorithms	Mean	Std	Best	Worst
FLAS	3.00E+03	5.7526	2997.0	3.03E+03
PSO_ELPM	9.63E+06	3.49E+06	3211.6	1.30E+07
HHO	5.13E+03	1.03E+03	3015.0	5.74E+03
DE	7.03E+05	3.83E+06	3029.4	2.10E+07
FLA	4.78E+06	4.53E+06	3082.1	1.11E+07
BWO	3.08E+03	31.694	3017.7	3.17E+03
RSO	9.80E+07	3.01E+06	8.76E+07	1.00E+08
RSA	3.28E+03	43.5377	3199.4	3.35E+03
TSA	3.04E+03	12.3493	3015.3	3.06E+03

**Table 9:** Optimal results for TBT.

Algorithms	Design Variable		Objective Function Value
	*x*_1_	*x*_2_	
FLAS	0.7861	0.4068	263.46355
PSO_ELPM	0.7868	0.4050	263.46389
HHO	0.7861	0.4069	263.46343
DE	0.7854	0.4094	263.4650
SCA	0.7871	0.4048	263.46364
FLA	0.7858	0.4081	263.46676
BWO	0.7575	0.4957	263.46431
RSO	0.7858	0.4240	264.15644
SMA	0.7861	0.4070	264.66549

**Table 10:** Statistical results of TBT.

Algorithm\Index	Mean	Std	Best	Worst
FLAS	263.3765	0.3850	263.46355	264.8725
PSO_ELPM	263.5856	0.1413	263.46389	264.0082
HHO	263.4989	0.6240	263.46343	263.7109
DE	263.5488	0.2119	263.4650	264.6178
SCA	264.7806	4.8423	263.46364	282.5938
FLA	265.2543	2.8059	263.46676	275.4833
BWO	263.7076	0.1671	263.46431	264.0034
RSO	270.5607	5.9360	264.15644	284.3287
SMA	268.8324	1.9769	264.66549	271.1422

**Table 11:** Design results of gear group.

Algorithm	Design Variable				Fitness Value
	*x*_1_	*x*_2_	*x*_3_	*x*_4_	
FLAS	19	17	50	44	2.70E-12
PSO_ELPM	19	16	50	44	2.70E-12
HHO	17	20	49	43	2.70E-12
DE	18	15	35	52	2.36E-09
SCA	31	13	54	52	2.70E-12
FLA	16	20	49	44	2.31E-11
BWO	12	13	28	38	2.70E-12
RSO	22	16	37	59	1.83E-08
SMA	19	17	43	50	3.07E-10
IGWO	19	17	50	44	2.70E-12

**Table 12:** Statistical results of design problems of gear group.

Algorithm\Index	Mean	Std	Best	Worst
FLAS	4.68E-10	8.22E-10	2.70E-12	3.30E-09
PSO_ELPM	7.42E-09	9.44E-09	2.70E-12	2.73E-08
HHO	2.29E-09	3.48E-09	2.70E-12	1.83E-08
DE	1.26E-07	2.25E-07	2.36E-09	9.98E-07
SCA	1.85E-09	1.60E-09	2.70E-12	6.51E-09
FLA	2.38E-09	2.64E-09	2.31E-11	1.31E-08
BWO	6.27E-09	8.07E-09	2.70E-12	2.73E-08
RSO	2.90E-03	5.50E-03	1.83E-08	2.42E-02
SMA	8.04E-09	8.90E-09	3.07E-10	2.73E-08
IGWO	4.31E-9	8.74E-09	2.70E-12	2.73E-08

**Table 13:** Optimization results of piston rod design.

Algorithm	Optimal Variable				Minimum Cost
	*x*_1_	*x*_2_	*x*_3_	*x*_4_	
FLAS	0.0500	0.7943	1.5876	500.0000	8.3410
PSO_ELPM	0.0500	0.7936	1.5854	500.0000	8.3630
HHO	0.0500	1.0891	2.1742	264.8728	9.3236
DE	0.0500	2.8864	2.1166	417.2231	7.9374
SCA	0.0530	0.7986	1.5862	500.0000	8.5800
FLA	0.0500	0.7986	1.5969	500.0000	8.5290
BWO	0.0500	0.8246	1.5942	494.8171	9.0300
RSO	0.0500	1.7789	2.1002	285.0632	30.076
SMA	0.0500	0.7923	1.5846	500.0000	8.6250
IGWO	0.0509	0.7937	1.5863	499.1632	8.3710

**Table 14:** Statistical results of piston rod design.

Algorithm	Mean	Std	Best	Worst
FLAS	0.8578	0.0163	8.341	0.8965
PSO_ELPM	6.7946	32.5143	8.3630	178.9467
HHO	260.4833	157.1277	9.3236	622.5394
DE	325.4745	348.3112	7.9374	1.47E+03
SCA	0.9368	0.05240	8.5800	1.0222
FLA	285.8016	338.5126	8.5290	1.26E+03
BWO	1.27E+00	0.4354	9.0300	2.71E+00
RSO	117.9851	200.9380	30.0760	1.02E+03
SMA	16.6741	48.3553	8.6250	159.3020
IGWO	31.0201	74.5183	8.3710	301.0950

**Table 15:** Results of design problems of gas transmission compressor.

Algorithms	Variable			Optimal Total Cost
	*x*_1_	*x*_2_	*x*_3_	
FLAS	53.4045	1.1898	24.7268	2,964,375.810043
PSO_ELPM	53.3761	1.1902	24.7375	2,964,378.339311
HHO	53.4352	1.1901	24.7191	2,964,375.922331
DE	53.6247	1.1905	24.6288	2,964,390.741786
SCA	54.3003	1.1926	24.927	2,964,439.591532
FLA	53.7641	1.1914	24.6519	2,964,386.475420
BWO	55.0000	1.1978	24.5709	2,964,545.887990
RSO	41.5644	1.1470	22.2803	2,977,866.495387
SMA	53.4466	1.1901	24.7186	2,964,415.619347
IGWO	51.1324	1.1340	37.1395	2,964,380.498417

**Table 16:** Statistical results of design problems of gas transmission compressors.

Algorithm\Index	Mean	Std	Best	Worst
FLAS	2.96E+06	25.4933	2,964,375.810043	2.96450E+06
PSO_ELPM	2.97E+06	3.83E+03	2,964,378.339311	2.978031E+06
HHO	2.96E+06	16.2642	2,964,375.922331	2.96453E+06
DE	2.96E+06	177.0009	2,964,390.741786	2.96535E+06
SCA	2.96E+06	340.4450	2,964,439.591532	2.96563E+06
FLA	2.96E+06	171.8834	2,964,386.475420	2.96534E+06
BWO	2.97E+06	7.17E+03	2,964,545.887990	3.00365E+06
RSO	3.05E+06	6.57E+04	2,977,866.495387	3.27322E+06
SMA	2.96E+06	5.94E-05	2,964,415.619347	2.96437E+06
IGWO	2.96E+06	138.0004	2,964,380.498417	2.96494E+06

**Table 17:** PVD results.

Algorithms	Design Variable				Fitness Value
	e_1_	e_2_	e_3_	e_4_	
FLAS	0.7873	0.3921	40.9869	191.0979	5925.7
PSO_ELPM	0.7984	0.4025	42.6198	170.4482	6075.4
HHO	0.8590	0.4270	44.8368	145.4212	6079.1
DE	0.9593	0.4253	49.2750	111.9953	6805.2
SCA	1.2994	0.2543	65.2013	10.4653	9012.2
FLA	0.8053	0.4019	40.9875	193.4983	6100.4
BWO	0.7553	0.4099	40.6985	197.0376	6095.6
RSO	0.7241	0.3423	53.2952	81.3113	10,771.0
SMA	0.7782	0.3847	40.3196	199.9997	5985.3
IGWO	0.8010	0.3979	41.5499	183.6757	5937.4

**Table 18:** PVD statistics.

Algorithms	Mean	Std	Best	Worst
FLAS	6846.7	575.8469	5925.7	7519.7
PSO_ELPM	6883.7	528.1608	6075.4	7788.2
HHO	6702.3	464.1332	6079.1	7507.1
DE	9993.3	1622.3000	6805.2	15,169.0
SCA	11,044.0	1094.5000	9012.2	11,697.0
FLA	19,821.0	32,279.0000	6100.4	170,480.0
BWO	6622.8	309.9841	6095.6	7281.4
RSO	53,919.0	82,523.0000	10,771.0	375,210.0
SMA	6021.0	361.1613	5985.3	7317.8
IGWO	7036.9	1037.4000	5937.4	9440.7

**Table 19:** Stepped cone pulley problem.

Algorithm	Variable					Minimum Weight
	*x*_1_	*x*_2_	*x*_3_	*x*_4_	*x*_5_	
FLAS	38.38439	52.85891	70.5076	84.5168	90.0000	16.5296
PSO_ELPM	20.5373	28.2978	50.9843	84.5432	90.0000	16.8305
HHO	20.7474	28.5272	50.8087	84.5149	89.9795	16.8268
DE	18.1514	28.2883	50.8292	84.9414	90.0000	16.9537
SCA	18.9110	28.6215	51.6447	86.1582	90.0000	17.1977
FLA	39.0286	29.5965	20.1708	49.0977	0.0540	17.0044
BWO	20.3360	28.7842	51.0233	84.8595	90.0000	16.9353
RSO	45.5075	55.7320	53.7411	75.4002	88.2041	17.1085
SMA	20.5423	28.2576	50.7969	84.4959	89.9998	16.8002
IGWO	2.9386	13.1223	32.9869	89.7359	88.1759	16.9241

**Table 20:** Statistical results of the stepped cone pulley problem.

Algorithm	Mean	Std	Best	Worst
FLAS	19.3022	2.4226	16.5296	26.9317
PSO_ELPM	10.1245	0.4252	16.8305	11.5840
HHO	9.9768	0.1607	16.8268	10.5558
DE	10.2580	0.1641	16.9537	10.6296
SCA	10.9584	0.3018	17.1977	11.6602
FLA	0.2474	0.2432	17.0044	1.0666
BWO	10.1119	0.1172	16.9353	10.3983
RSO	2.57E+03	1.35E+03	17.1085	6.17E+03
SMA	9.8007	4.57E-04	16.8002	9.8026
IGWO	10.4245	0.2178	16.9241	10.8051

**Table 21:** Comparison and analysis of SDM results.

I Parameter	SDM	
	*Lb*	*Ub*
*I_ph_*	0	1
*I_sd_*	0	1
*R_s_*	0	0.5
*R_sh_*	0	100
*n*	1	2

**Table 22:** Experimental results of FLAS algorithm on SDM.

Serial Number	Actual Data			Algorithmic Estimation Data		
	*I*	*V*	*P*	*I_m_*	*IAE_I_*	*IAE_P_*
1	0.7640	−0.2057	−1.572E-01	7.64E-01	1.51E-04	3.10E-05
2	0.7620	−0.1291	−9.837E-02	7.62E-01	4.91E-04	6.34E-05
3	0.7605	−0.0588	−4.472E-02	7.61E-01	7.44E-04	4.38E-05
4	0.7605	0.0057	4.335E-03	7.60E-01	4.00E-04	2.28E-06
5	0.7600	0.0646	4.910E-02	7.59E-01	9.47E-04	6.12E-05
6	0.7590	0.1185	8.994E-02	7.58E-01	9.14E-04	1.08E-04
7	0.7570	0.1678	1.270E-01	7.57E-01	1.76E-04	2.95E-05
8	0.7570	0.2132	1.614E-01	7.56E-01	7.40E-04	1.58E-04
9	0.7555	0.2545	1.923E-01	7.55E-01	2.70E-04	6.88E-05
10	0.7540	0.2924	2.205E-01	7.54E-01	1.85E-04	5.41E-05
11	0.7505	0.3269	2.453E-01	7.52E-01	1.02E-03	3.35E-04
12	0.7465	0.3585	2.676E-01	7.47E-01	9.38E-04	3.36E-04
13	0.7385	0.3873	2.860E-01	7.40E-01	1.61E-03	6.22E-04
14	0.7280	0.4137	3.012E-01	7.27E-01	7.01E-04	2.90E-04
15	0.7065	0.4373	3.090E-01	7.07E-01	2.47E-04	1.08E-04
16	0.6755	0.4590	3.101E-01	6.75E-01	4.87E-04	2.23E-04
17	0.6320	0.4784	3.023E-01	6.31E-01	1.40E-03	6.71E-04
18	0.5730	0.4960	2.842E-01	5.72E-01	1.13E-03	5.61E-04
19	0.4990	0.5119	2.554E-01	4.99E-01	4.17E-04	2.14E-04
20	0.4130	0.5265	2.174E-01	4.14E-01	5.80E-04	3.05E-04
21	0.3165	0.5398	1.708E-01	3.17E-01	9.40E-04	5.07E-04
22	0.2120	0.5521	1.170E-01	2.12E-01	3.90E-04	2.15E-04
23	0.1035	0.5633	5.830E-02	1.03E-01	5.20E-04	2.93E-04
24	−0.0100	0.5736	−5.736E-03	−9.12E-03	8.82E-04	5.06E-04
25	−0.1230	0.5833	−7.175E-02	−1.24E-01	1.48E-03	8.64E-04
26	−0.2100	0.5900	−1.239E-01	−2.10E-01	4.75E-04	2.80E-04

**Table 23:** Comparison of different algorithms on SDM.

Algorithm	FLAS	FLA	DMOA	IGWO	ISSA	CSA	SCHO	BWO	TSA
*I_ph_*	0.760689	0.760385	0.760556	0.750763	0.760528	0.760608	0.760424	0.762818	0.762663
*I_o_*	0.505267	0.323282	0.437245	0.739541	0.239303	0.310693	0.540756	0.454039	0.405659
*R_sh_*	66.009368	67.473579	60.407287	58.065965	52.288364	52.889838	73.901078	38.757679	67.221903
*R_s_*	0.034357	0.037441	0.034849	0.030098	0.037780	0.031066	0.033881	0.037427	0.036881
*n*	1.527676	1.481111	1.512398	1.571048	1.451453	1.604563	1.534808	1.518459	1.504539
*RMSE*	1.096E-03	1.396E-03	1.871E-03	1.625E-03	3.852E-03	2.051E-03	1.242E-03	5.030E-03	2.455E-03

## Data Availability

All data generated or analyzed during the study are included in this published article.
